# Ligand binding to a Ni–Fe cluster orchestrates conformational changes of the CO-dehydrogenase–acetyl-CoA synthase complex

**DOI:** 10.1038/s41929-025-01365-y

**Published:** 2025-07-11

**Authors:** Jakob Ruickoldt, Julian Kreibich, Thomas Bick, Jae-Hun Jeoung, Benjamin R. Duffus, Silke Leimkühler, Holger Dobbek, Petra Wendler

**Affiliations:** 1 Department of Biochemistry, Institute of Biochemistry and Biology, University of Potsdam, Potsdam-Golm, Germany; 2 Department of Biology, Humboldt-Universität zu Berlin, Berlin, Germany; 3 Department of Molecular Enzymology, Institute of Biochemistry and Biology, University of Potsdam, Potsdam-Golm, Germany

**Keywords:** Cryoelectron microscopy, Biocatalysis, Enzyme mechanisms

## Abstract

Catalytic metal clusters play critical roles in important enzymatic pathways such as carbon fixation and energy conservation. However, how ligand binding to the active-site metal regulates conformational changes critical for enzyme function is often not well understood. One carbon fixation pathway that relies heavily on metalloenzymes is the reductive acetyl-coenzyme A (acetyl-CoA) pathway. In this study, we investigated the catalysis of the last step of the reductive acetyl-CoA pathway by the CO-dehydrogenase (CODH)–acetyl-CoA synthase (ACS) complex from *Carboxydothermus hydrogenoformans*, focusing on how ligand binding to the nickel atom in the active site affects the conformational equilibrium of the enzyme. We captured six intermediate states of the enzyme by cryo-electron microscopy, with resolutions of 2.5–1.9 Å, and visualized reaction products bound to cluster A (an Ni,Ni-[4Fe4S] cluster) and identified several previously uncharacterized conformational states of CODH–ACS. The structures demonstrate how substrate binding controls conformational changes in the ACS subunit to prepare for the next catalytic step.

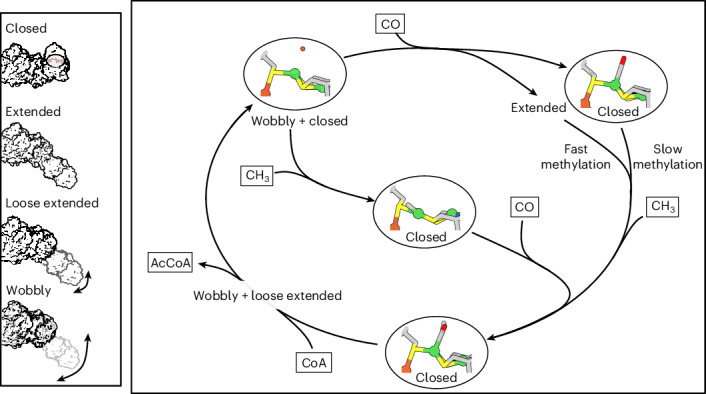

## Abstract (web summary)

The CO dehydrogenase–acetyl-coenzyme A synthase complex produces acetyl-coenzyme A from CO_2_, but its structural dynamics during catalysis remain unresolved. Now cryo-EM maps of six intermediate states reveal how ligand binding to a Ni–Fe cluster orchestrates the conformational changes of the complex during catalysis.

## Main

CO-dehydrogenase (CODH)–acetyl-coenzyme A synthase (ACS) is an enzyme complex in the reductive acetyl-coenzyme A (acetyl-CoA) pathway that reduces two molecules of CO_2_ to acetyl-CoA. In a fossil fuel-free future, feedstock chemicals and fuels will have to be synthesized from CO_2_. Thus, mimicking one of the oldest^1–3^ and most energy-efficient biological strategies to fixate CO_2_, the reductive acetyl-CoA pathway^4,5^, might be a promising way to achieve this. Indeed, for a few years, this process has been employed commercially to produce ethanol from syngas using the bacterium *Clostridium autoethanogenum*^6^.

**Fig. 1:**
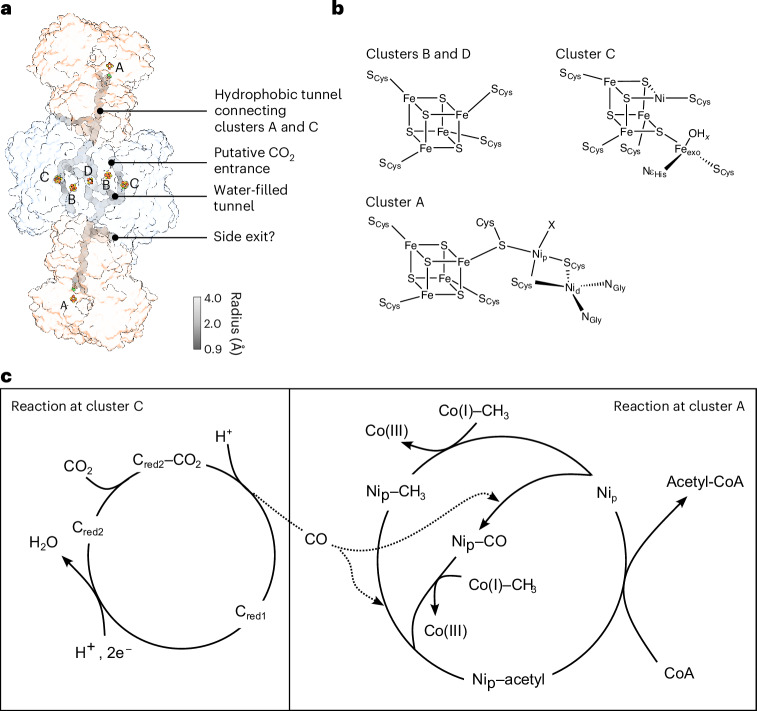
The bacterial CODH–ACS class 1 complex, its clusters and the proposed reaction cycles. **a**, The overall structure of CODH–ACS from *C. hydrogenoformans* (Protein Data Bank (PDB) no. 7ZKJ) in surface representation. CODH is coloured blue and ACS is coloured red. The metal clusters A–D are shown as spheres. The tunnel system was calculated with a probe radius of 0.9 Å and is coloured according to the colour code bar. The proposed function of the different parts of the tunnel system are indicated on the right. Note that the complex has *C*_2_ symmetry. **b**, Structural formulae of clusters A–D. CO_2_ is activated by binding to the Ni and in exo Fe (Fe_exo_) of cluster C. The ligand X in cluster A denotes the varying ligand at the proximal Ni (Ni_p_) of cluster A. The distal Ni (Ni_d_) has mainly a structural role as its oxidation state is unchanged during catalysis. NεHis refers to the side chain histidine N atom. **c**, Proposed reaction cycle. CO_2_ binds at cluster C in the fully reduced state (C_red2_). Concomitant with the reduction to CO, cluster C is oxidized to the one-electron reduced C_red1_ state. After two-electron reduction and protonation, C_red2_ is recovered. Note that the exact order of protonation and substrate release is not clear. The CO formed is then transported to cluster A through a hydrophobic tunnel. At cluster A, CO condenses with a methyl moiety to form a Ni–acetyl intermediate. CO and the methyl moiety (carried by a Co atom in a corrinoid cofactor) can bind to Ni_p_ in random order. The Ni–acetyl intermediate is then thiolysed by CoA, closing the catalytic cycle.

The reductive acetyl-CoA pathway consists of two branches^7^. In the methyl branch, CO_2_ is reduced via formate to a methyl moiety carried by the corrinoid iron–sulfur protein (CoFeSP). In the carbonyl branch, CO_2_ is reduced to CO by CODH. Finally, the two branches merge through the action of ACS, which catalyses the condensation of CO, the CH_3_ moiety and CoA to yield acetyl-CoA. In bacteria, the homodimeric CODH forms a complex with two ACS subunits and the CO is transported from the active site of CODH (cluster C) to the active site of ACS (cluster A, an Ni,Ni-[4Fe4S] cluster) through a 70-Å-long hydrophobic proteinaceous tunnel^8–10^ (Fig. 1). Furthermore, evolution has yielded two classes of bacterial CODH–ACS complexes that differ in their subunit arrangement^11^. The first class includes the enzymes from *Moorella thermoacetica* and *Carboxydothermus hydrogenoformans*, while the second class, discovered later, includes the enzyme from *C. autoethanogenum*^12^. In this study, we investigated the CODH–ACS complex of *C. hydrogenoformans*.

The ACS subunit can adopt several conformations. Two conformations have been studied at high resolution by X-ray crystallography^9,13^. In the open conformation, cluster A is exposed to solvent and access to the CO-transporting tunnel is restricted. In the closed conformation, solvent cannot access cluster A, but the tunnel is open. Conformations that are even more extended than the open conformation have recently been observed by negative stain electron microscopy of *M. thermoacetica* CODH–ACS (ref. ^14^). The factors that influence the conversion and equilibrium of these conformations are currently unknown. An obvious assumption would be that substrate binding to different active sites modulates the conformational landscape, although this has not yet been verified.

ACS binds substrates at cluster A. Cluster A is a [4Fe4S] cluster connected by a cysteine to two Ni atoms that are bridged by two further cysteines. The Ni ion proximal (proximal Ni, Ni_p_) to the [4Fe4S] cluster is the site of catalysis, while the distal Ni ion most probably plays a structural role as its oxidation state is unchanged during catalysis (Fig. 1). The reaction involves the random addition of CO or a CH_3_ moiety to cluster A, followed by addition of the second substrate^15^. The CO and CH_3_ moiety then condense to form Ni–acetyl, which is thiolysed by CoA, closing the cycle. A change in the cluster geometry might trigger greater movement of the protein. The geometry of cluster A in various substrate-bound states has been investigated by X-ray absorption spectroscopy, extended X-ray absorption fine structure (EXAFS) spectroscopy^16,17^, electron paramagnetic resonance spectroscopy^18^ and X-ray crystallography^19^. The carbonylated state is thought to be tetrahedral^19^, while the methylated and acetylated states are proposed to be square planar^16,17^.

The precise conformational changes that occur in CODH–ACS during the catalytic cycle are currently not known. In this study, we analysed the catalytic cycle of CODH–ACS by cryo-electron microscopy (cryo-EM) after trapping six intermediate states of the reaction. We found that ACS responds conformationally to the ligand bound to cluster A: methylation favours a closed state with an open tunnel system, carbonylation also favours this state and a methylation-ready extended state, while the ACS is mostly in the closed state upon acetylation. CoA then probably disrupts the closed state. These findings led us to propose a revised catalytic cycle for ACS.

## Results

### ACS of the as-isolated CODH–ACS shows high flexibility

**Fig. 2:**
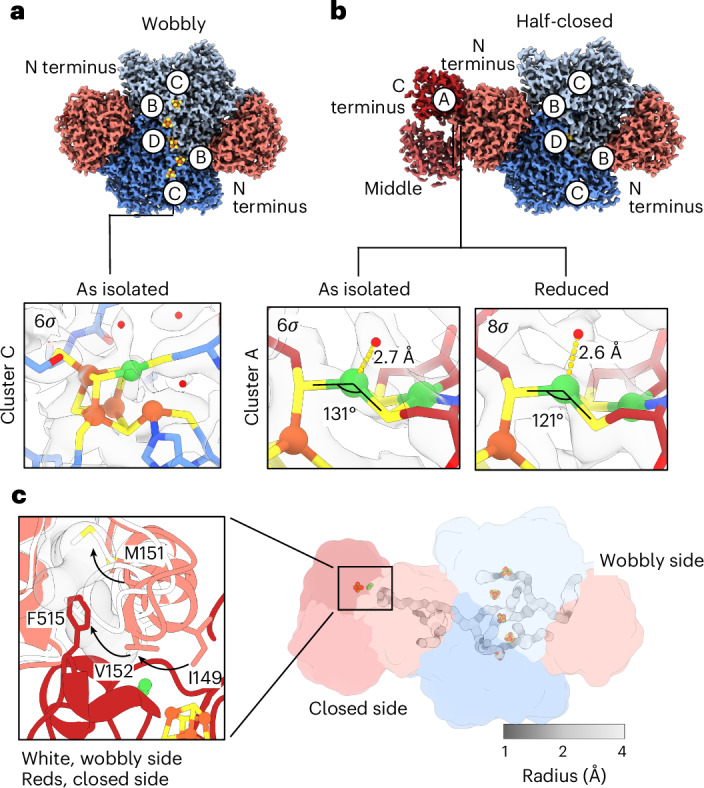
Cryo-EM structures of the as-isolated and reduced CODH–ACS. **a**,**b**, Two predominant species were identified in the datasets, which we denote as wobbly (**a**) and half-closed (**b**). In **a**, the density of the wobbly state shows CODH subunits (blue and light blue) and the ACS N-terminal domain (salmon). The middle and C-terminal domains are not visible in this map. In **b**, the half-closed state shows the middle and C-terminal domains on the left side (light red and red, respectively). In **a** and **b**, the metal clusters are shown as spheres coloured blue (N), red (O), yellow (S), orange (Fe) and green (Ni). The lower panels show a close up of the Coulombic potential maps around cluster C (wobbly) and cluster A (half-closed) contoured at the indicated standard deviation (grey surface). Water molecules are shown as red spheres. Relevant distances and angles are indicated. **c**, Tunnel system of CODH–ACS in the half-closed state. The tunnels were calculated using Caveranalyst 2.0 and are coloured according to their radii. Inset: a close-up of the tunnel at cluster A and superposition of the structure of the ACS N-terminal domain in the two states. The N-terminal domain of the wobbly side (white) and the closed side are shown in cartoon representation (coloured as in **b**). Note that the tunnel directly leading to cluster A was drawn manually and not calculated by Caveranalyst (as Caveranalyst only finds tunnels leading to the protein surface not between active sites of a protein). Crucial residues are shown as stick structures. The clashes of Ile149 with cluster A and of Val152 with Phe515 seem to prevent the ‘snapping’ movement of the gating helix (indicated by the arrows), through which Met151 sticks into the tunnel and seals it.

**Fig. 3:**
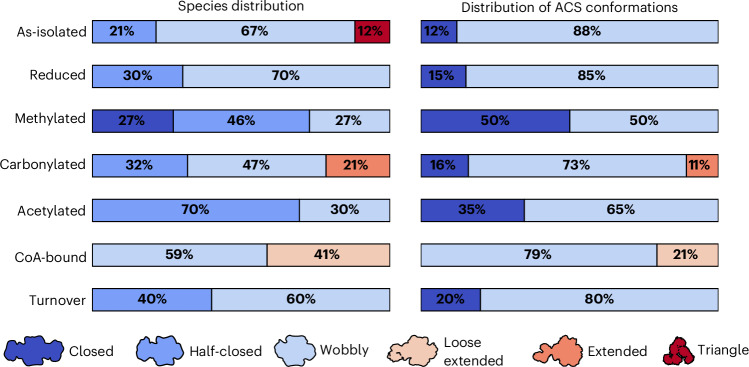
Distribution of species and ACS conformations in the different datasets. The percentage distributions were calculated from the particles found in the initial template pick. The distribution of ACS conformations was calculated from the species distribution by counting two closed conformations for the closed state, one closed and one wobbly conformation for the half-closed state and so on. The triangle state was not considered for the ACS conformations as it is not physiologically relevant and is probably an artefact of the interaction of the ACS-6xHis tag with the Ni_p_.

**Extended Data Fig. 1:**
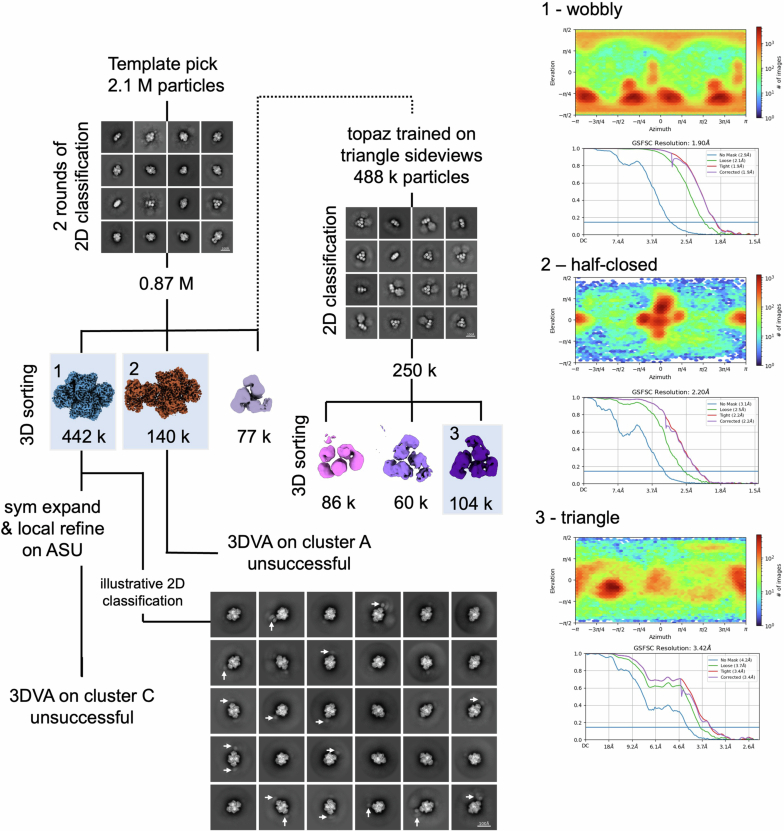
Overview of the particle picking, sorting and 3D reconstruction work flow for the as isolated data set. Templates for the initial template pick were generated by blob picking the reduced data set followed by 2D classification generating the templates. Template picked particles were sorted in two stages. First, by 2D classification and second in the three-dimensional space by ab-initio reconstruction with several classes followed by heterogenous refinement. We performed a 2D classification on the wobbly particles (limited to 12 Å, 400 classes) illustrating the conformational flexibility of ACS. Selected front views are shown at the bottom. Arrows mark the densities for the middle and C-terminal ACS domains. Solid lines indicate particle flow and dotted lines the use of particles for training a Topaz picking model. Abbreviations: ASU- asymmetric unit, 3DVA - 3D variability analysis.

Three main species were found in untreated as-isolated CODH–ACS that are denoted wobbly, half-closed and triangle based on the arrangement of the ACS domain (Figs. 2 and 3, Extended Data Fig. 1 and Supplementary Fig. 1). The most dominant species is the wobbly state, in which only the CODH core and the N-terminal domains (residues 2–315) of the bound ACS subunits are visible (Fig. 2a). This conformation probably represents the energetically favoured state of the ACS in solution. The structure of this state was determined at a resolution of 1.9 Å, exploiting the *C*_2_ symmetry of the complex. In the half-closed state, one ACS is in the closed conformation known from the crystal structures, while the other ACS is in a wobbly conformation (Fig. 2b). On the wobbly side, the N-terminal ACS domain resembles that of the crystal structure of the open state. The triangle state is an artefact due to the binding of the 6xHis tag to cluster A (Supplementary Discussion).

In the wobbly state, the tunnel connecting cluster C and cluster A is closed by a gating helix containing residues 140–153 (Fig. 2 and Supplementary Fig. 2). Met151 in the N-terminal ACS domain serves as a plug to seal the tunnel to cluster C, as also observed in the open conformation of ACS^9,13^. While we observed an unresolved density for the middle and C-terminal ACS domains in two-dimensional (2D) class averages (Extended Data Fig. 1), we did not detect this density in three-dimensional (3D) reconstructions, probably due to their high flexibility. This flexibility is independent of the protein density on the grids as we observed the same behaviour in negative stain electron microscopy (EM) and cryo-EM of diluted samples (Supplementary Fig. 3). We did not observe the open conformation that was previously found in crystal structures of ACS, but this might be a substate of the wobbly state, that allows the formation of a crystal lattice^9,13^.

The structure of the half-closed state was determined at 2.2 Å resolution to allow analysis of the density around cluster A. The conformation on the closed side is similar to that found in the crystal structure (root mean squared deviation (RMSD) for Cα atoms is 0.55 Å (ref. ^20^)). Here, the tunnel is opened by the gating helix in the N-terminal ACS domain, which constitutes the only conformational difference between the closed and wobbly conformations. Most of the residues in this helix are evolutionarily conserved: in the closed conformation, Phe515 and Ni_p_ block the space occupied by Val152 and Ile149 in the wobbly state (Fig. 2c and Supplementary Fig. 2). Val152 is highly conserved and residues of similar size (Ile, Leu, Val and Thr) are found at position 149. Furthermore, we observe several interactions between the N-terminal domain and the C-terminal and middle domains in the closed conformation that stabilize the closed conformation (Fig. 2c and Supplementary Fig. 2). The gating helix interacts with Glu332 in the middle domain via Lys150 and with the backbone oxygen of Gly599 near cluster A in the C-terminal domain via Arg145 (Supplementary Fig. 2c). These interactions position the gating helix in such a way that the hydrophobic tunnel is open. Furthermore, a highly conserved Gly148 is positioned in the middle of the gating helix. This Gly148 introduces a break in the helix, thereby possibly acting as a hinge and enabling a snapping motion if the C-terminal and middle domains are not present to stabilize the closed conformation (Supplementary Fig. 2). Then, in the wobbly state, Trp154 slips into a binding pocket where it interacts via π–π bonding with Arg250. Met151 serves as a plug for the CO-transporting tunnel. In a previous study, we found that the F515A mutation did not cause major CO leakage^11^. This is understandable in view of our current data as Phe515 does not close the tunnel, but opens it. Surprisingly, we found no structural differences between the wobbly and closed conformations at the interface between the N-terminal ACS domain and CODH. This indicates that the conformational states of ACS are transmitted to the CODH only by the opening and closing of the tunnel and not through long-range domain interactions between cluster A and cluster C.

In the as-isolated state, the oxidation state of the clusters in the CODH–ACS complex is not strictly defined. The enzyme was treated with dithionite (midpoint potential *E*_m_ ≈ −550 mV versus the normal hydrogen electrode (NHE) at pH 7.6 and 1 mM dithonite^21^) during cell lysis, but was oxidized by the unavoidable residual oxygen content in the anoxic boxes during purification. Similar to almost all CODHs, its cluster C must be reductively activated to the C_red1_ state capable of CO oxidation (*E*_m_ ≈ −150 mV versus NHE (ref. ^22^)). This indicates that cluster C is mainly in the C_ox_ state. Furthermore, the reduction potentials of the reduced states of clusters A, B and D are even more negative than that of C_red1_ (ref. ^23^). Thus, they are mainly oxidized.

### Reduction does not induce conformational changes

**Extended Data Fig. 2:**
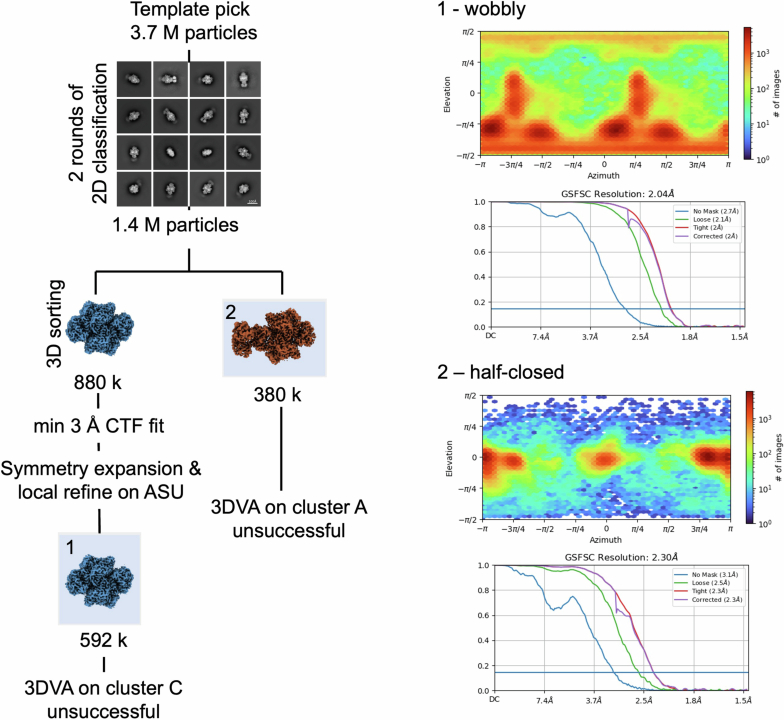
Overview of the particle picking, sorting and 3D reconstruction work flow for the reduced data set. Templates for the initial template pick were generated by blob picking the reduced data set followed by 2D classification generating the templates. Template picked particles were sorted in two stages. First, by 2D classification and second in the three-dimensional space by ab-initio reconstruction with several classes followed by heterogenous refinement. Abbreviations: ASU - asymmetric unit, 3DVA - 3D variability analysis.

Upon incubation with the strong reductant Ti(III)–EDTA (−435 mV versus NHE at pH 7.2, capable of reducing CO_2_ to CO (ref. ^20^)), a decrease in absorbance at 420 nm, typical of the reduction of [4Fe4S] clusters, was observed (Supplementary Fig. 4). In this reduced state, only the wobbly and half-closed states were found in cryo-EM experiments. The structures were determined at 2.04 and 2.3 Å resolution for the wobbly and half-closed states, respectively (Extended Data Fig. 2). While the overall structure of the complex matches that in the as-isolated state (RMSD for Cα atoms is 0.3 Å in the half-closed state), the ratio of half-closed to wobbly particles changed from 1:3 to ~1:2. To quantify the examiner bias on the percentages of the species and thus their significance, the as-isolated dataset was analysed by three independent examiners. The observed differences in populations were at most ~5% (Supplementary Fig. 5). Thus, the reduction of cluster A did not significantly alter the equilibrium distribution between the wobbly and closed states (Fig. 3). The triangle state was not found in the reduced state, which could be due to the reduced affinity of Ni ions to histidine after reduction.

Although the obtained resolutions should allow a comparison of cluster C at the atomic level, we could not analyse it as cluster C in the sample was not sufficiently mature. Ni refined only to an occupancy of 12–15%. However, the heterologously produced CODH–ACS sample had ~25% of the CO oxidation activity of the enzyme (Supplementary Fig. 4) directly purified from *C. hydrogenoformans* (400 U mg^−1^ (ref. ^13^)). Therefore, we used cluster C for the in situ generation of CO and focused our analysis on cluster A in the ACS subunit.

Cluster A seems to have similar geometries in the as-isolated and reduced states. It adopts a slightly distorted tetrahedral coordination with density above the Ni_p_, which we modelled as water or hydroxide in accord with a recent EXAFS study^16^. However, the as-isolated map showed some anisotropy (Supplementary Fig. 6), so the geometry could not be determined with complete certainty. Cluster A can be poisoned by trace amounts of Zn displacing the Ni_p_ (ref. ^24^). Zn^2+^ at the proximal position is catalytically inactive, tetrahedrally coordinated and probably binds water or hydroxide similarly to Ni. The sample used contains about eight Ni and around one Zn atom per CODH–ACS protomer (Extended Data Table 3 and Supplementary Fig. 7), indicating that the ions are bound not only to the active sites but also to the protein surface. In a previous study, we obtained an occupancy of Ni_p_ of 38 ± 9% (*n* = 5) by anomalous X-ray scattering, allowing the metal content in the active site to be determined^11^. In this study, we followed the same purification protocol and obtained a similar activity in acetyl-CoA synthesis and thus expect a similar Ni occupancy. How Zn-containing ACS affects the results is discussed in more detail in the Supplementary Discussion. If we now turn to the catalytic cycle, the next catalytic step at cluster A is the binding of either CO or a methyl moiety.

### Methylation of cluster A favours the closed conformation

The methylation of cluster A by methyl cobinamide is highly favourable (*K*_eq_ = 341; Supplementary Fig. 4), ensuring a saturation of more than 99% at the concentration used for cryo-EM (0.5 mM). Although the native substrate for methylation is CH_3_–CoFeSP, we used methyl cobinamide as the micrographs were too crowded by CH_3_–CoFeSP to allow single particle analysis when using appropriate concentrations for complex formation (Supplementary Fig. 8).

The CODH–ACS complex changed shape upon methylation of cluster A: the wobbly state is much less populated and the half-closed state is the dominant species (Fig. 3). The structures of the wobbly and half-closed states were determined at resolutions of 2.23 and 2.26 Å, respectively. Furthermore, a fully closed state is observed, which could be reconstructed to a resolution of 2.35 Å applying *C*_2_ symmetry. Altogether, ~50% of the ACS subunits are in the closed conformation under methylating conditions. If we assume the worst case that 15% of the ACS subunits in the closed conformation found in the reduced dataset contain Zn, ~70% of the ACS in the closed conformation observed after methylation would still contain Ni. Therefore, the Ni content in this species is between 70% and 100% at the proximal site.

**Fig. 4:**
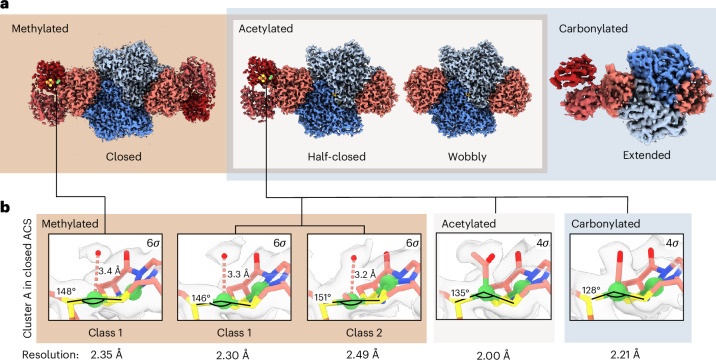
Cryo-EM structures of CODH–ACS in the methylated, acetylated and carbonylated states. **a**, Density maps of methylated, acetylated and carbonylated CODH–ACS. The half-closed and wobbly states are observed in all conditions, the closed state only after methylation and the extended state only after carbonylation. **b**, Cluster A in the closed and half-closed states. The resolution in the extended state did not allow an analysis of cluster A. Densities are contoured at the indicated standard deviation (grey surface). Relevant distances and angles are indicated. The metal clusters are shown as spheres coloured blue (N), red (O), yellow (S), orange (Fe) and green (Ni). Water molecules are shown as red spheres.

**Extended Data Fig. 3:**
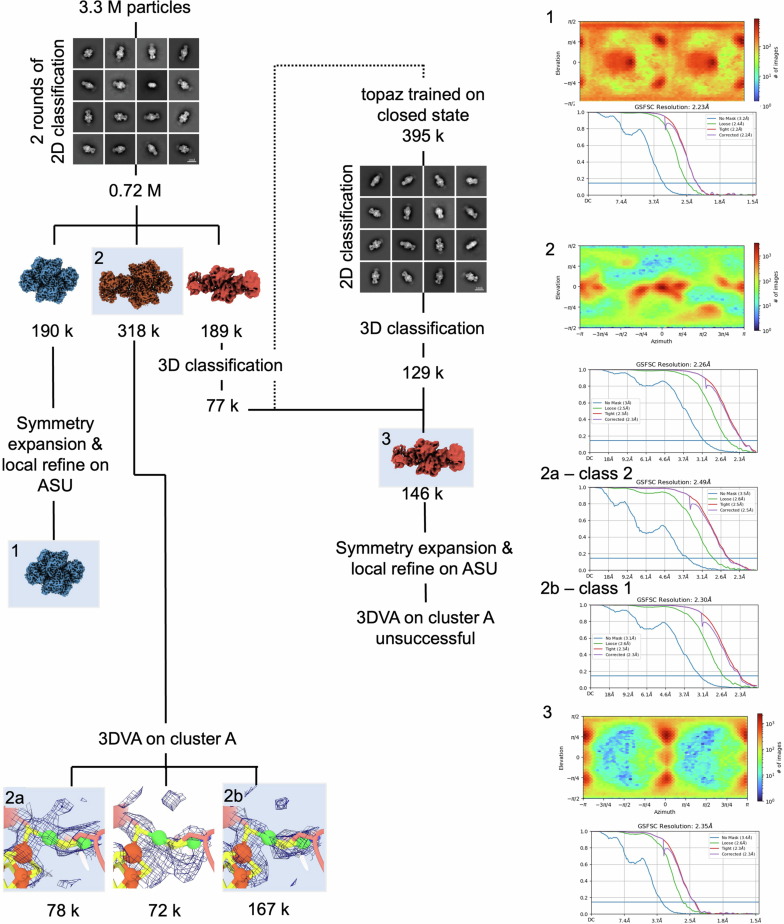
Overview of the particle picking, sorting and 3D reconstruction work flow for the methylated data set. Templates for the initial template pick were generated by blob picking the reduced data set followed by 2D classification generating the templates. Template picked particles were sorted in two stages. First, by 2D classification and second in the three-dimensional space by ab-initio reconstruction with several classes followed by heterogenous refinement. Straight lines indicate the flow of particles and dotted lines the use of particles for the training of a Topaz picking model. Abbreviations: ASU - asymmetric unit, 3DVA - 3D variability analysis.

After methylation, we observed heterogeneity of the half-closed state in cluster A and resolved it by 3D variability analysis, obtaining two relevant classes (Fig. 4, Extended Data Fig. 3 and Supplementary Fig. 9). Class 1 was found in both the half-closed and closed states and was reconstructed to a resolution of ~2.3 Å (a similar local resolution was determined at cluster A, Supplementary Fig. 10). Here, Ni_p_ has a distorted trigonal coordination and the density is located above Ni_p_ (Fig. 4 and Supplementary Fig. 9). We modelled this density as water, but it is too far away to be a ligand of Ni_p_ (>3.3 Å). This density might arise from a subset of particles in which Zn occupies the proximal position with a tetrahedrally ligated water. In Class 2, apical density is also observed as well as weak density in the axial position, completing the square-planar conformation. For illustrative purposes, we modelled the methyl moiety, which refined to a distance of 1.78 Å from Ni_p_, similar to the distance of 1.98 Å for the Ni–CH_3_ bond determined by EXAFS^16^. This ligand seems to push Ile149 out of the void space (Supplementary Fig. 9b). We suspect that the weak density of the methyl moiety is due to the rather low resolution of the EM map of this class (2.49 Å) and the clash with Ile149, which might increase the flexibility of the methyl moiety and thus decrease its detectability.

### Carbonylation favours the closed and extended state

**Fig. 5:**
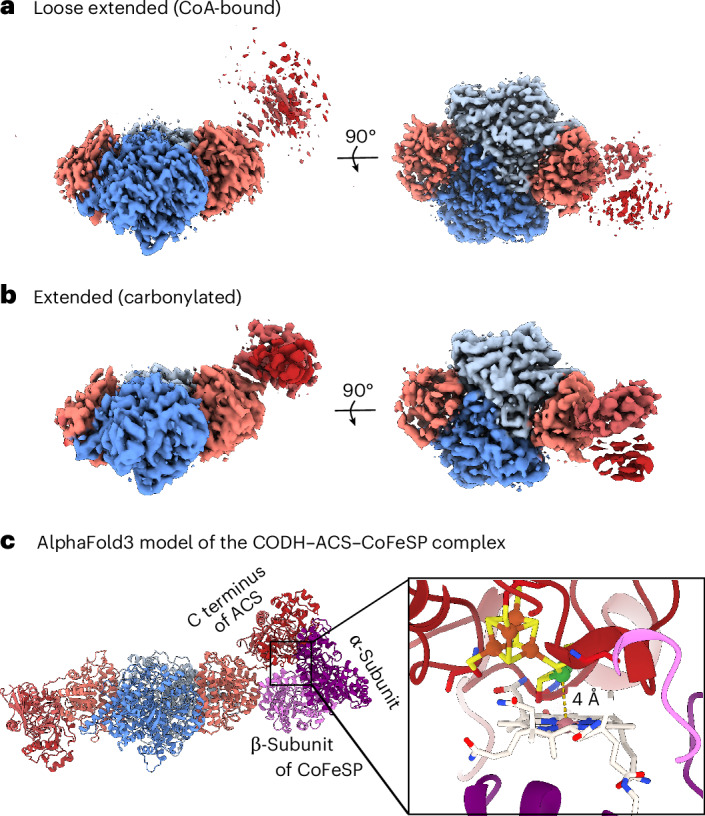
Cryo-EM structures of the extended and loose extended states found in the presence of CO and CoA, respectively. **a**,**b**, 3DFlex consensus maps (5*σ*) of the loose extended state found in the presence of CoA (**a**) and of **t**he extended state found in the carbonylated dataset (**b**). The consensus map only resolves low-resolution features of the extended state. **c**, AlphaFold3 model of the CODH–ACS–CoFeSP complex. The conformation of CODH–ACS matches the map of the extended state. Inset: a close-up of the interface of the C-terminal domain of ACS and CoFeSP. The corrinoid cofactor and cluster A are at a distance suitable for methyl transfer. In **a**–**c**, CODH is coloured blue and ACS is coloured red. In c, CoFeSP is coloured purple (α-subunit) and pink (β-subunit).

**Extended Data Fig. 4:**
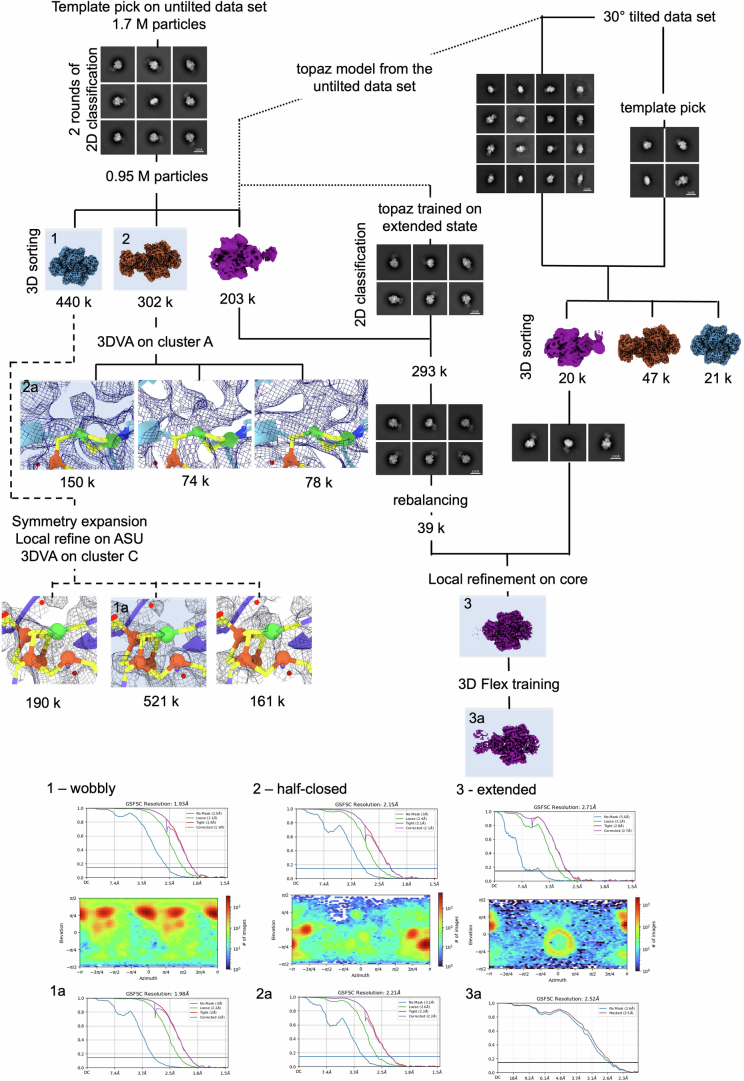
Overview of the particle picking, sorting and 3D reconstruction work flow for the carbonylated data set. Templates for the initial template pick were generated by blob picking the reduced data set followed by 2D classification generating the templates. Template picked particles were sorted in two stages. First, by 2D classification and second in the three-dimensional space by ab-initio reconstruction with several classes followed by heterogenous refinement. Straight and broken lines indicate the flow of particles and dotted lines the use of particles for the training of a Topaz picking model. Abbreviations: ASU - asymmetric unit, 3DVA - 3D variability analysis.

We generated CO in situ by incubating CODH–ACS with CO_2_ and Ti(III)–EDTA overnight, yielding around 60 molecules of CO per CODH monomer (corresponding to ~200 µM CO, see Methods for the calculation). CO generated at cluster C can reach cluster A both through the tunnel or via solution as ACS has a very high affinity for CO (*K*_d_ = 0.74 µM (ref. ^11^)) and is able to outcompete haemoglobin for CO even if the tunnel is blocked^11^. The half-closed, wobbly and a new state, called the extended state, are found in this CO-exposed sample (Figs. 3–5 and Extended Data Fig. 4). In the half-closed state (resolution 2.21 Å), apical density is present above Ni_p_, which we modelled as CO. Ni_p_ has a tetrahedral conformation and the Ni–CO bond length refined to 1.56 Å, shorter than the values derived from quantum mechanics/molecular mechanics^25^, density functional theory (DFT) and EXAFS^17,26^ (bond lengths of 1.7–1.8 Å). However, this difference could be due to the non-atomic resolution (Fig. 4). Furthermore, we propose to have identified CO molecules in the hydrophobic tunnel. Their positions coincide with the xenon binding sites found for the CODH–ACS of *M. thermoacetica*^10^ (Supplementary Fig. 11). Xenon is hydrophobic, has a similar cross-section to CO and can be identified by anomalous scattering^27^. It seems that CO_2_ reduction did not stop after saturating cluster A, but continued at least until the tunnel was filled. However, cluster A cannot be methylated in the closed conformation. Therefore, carbonylated ACS in the closed conformation might be a detour in the catalytic cycle.

The structure of the wobbly state of the carbonylated sample was determined at a resolution of 1.98 Å and was identical to those discussed above. In the extended state, the CODH subunit and ACS N-terminal domain are in the same conformation as in the wobbly state. We observed some anisotropy in the map of the initial dataset, which we could efficiently lower by collecting a dataset at a tilt angle of 30°. Furthermore, we observed flexibility of the middle and C-terminal ACS domain, which we resolved best with 3D flexible refinement (Supplementary Fig. 12 and Supplementary Video 1), but it prevented us from obtaining a map at sufficient resolution to analyse the binding state at cluster A (Fig. 5 and Supplementary Figs. 12 and 13). The extended state of the carbonylated sample is similar to the extended and hyperextended states found by Cohen et al. for the CODH–ACS of *M. thermoacetica*^14^ by negative stain EM (Supplementary Fig. 14) and shows a larger rearrangement compared with the closed and open states of ACS (Supplementary Figs. 13 and 15 and Supplementary Video 1). Despite the rather low resolution of the cryo-EM map, we could trace the domain arrangement of ACS in this map. Surprisingly, an AlphaFold3 model^28^ of the ACS–CoFeSP complex shows a very similar arrangement (Fig. 5c). In this model, the ACS C-terminal domain is bound to the C-terminal domain of the α-subunit of CoFeSP carrying the corrinoid cofactor. The N-terminal domain of the α-subunit of CoFeSP binds the middle domain of ACS, while the N-terminal domain of ACS is in contact with the β-subunit of CoFeSP. This fits with a previous model, where the C-terminal domain of the α-subunit is thought to act as a mobile element managing the interactions with ACS and methyl transferase^29^. The conformation of CoFeSP in the AlphaFold3 model closely resembles that in the methylation-ready state found in the complex with folate-bound methyltransferase^27^ and is similar to the conformation of CoFeSP complexed with its activator^30^. Furthermore, such a conformation was recently observed in the distantly related CODH–ACS of *C. autoethanogenum* complexed with CoFeSP^31^.

We suspect that the tunnel is closed in the extended state as it is kept open by the interaction of the gating helix with the C-terminal and middle domains, which are not in contact here. How the binding of CO triggers that conformational change remains unclear.

### Acetylation favours a tetrahedral configuration and the closed state

**Extended Data Fig. 5:**
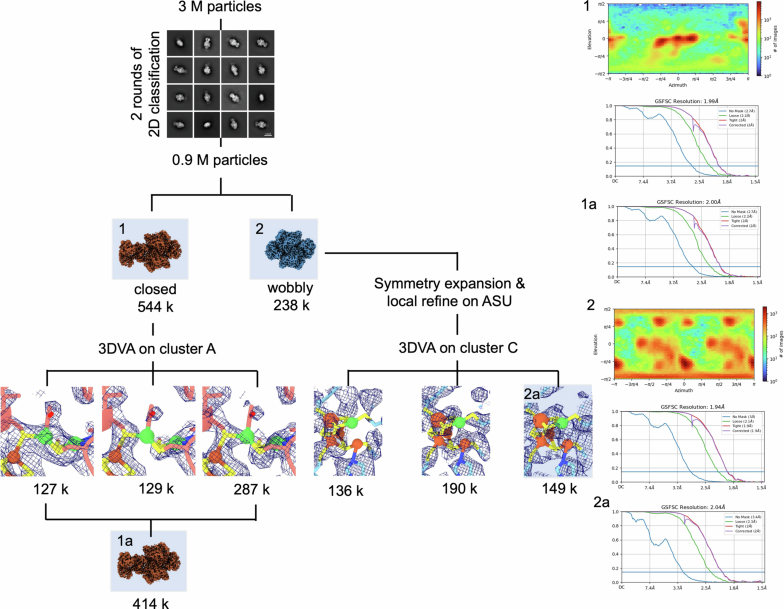
Overview of the particle picking, sorting and 3D reconstruction work flow for the acetylated data set. Templates for the initial template pick were generated by blob picking the reduced data set followed by 2D classification generating the templates. Template picked particles were sorted in two stages. First, by 2D classification and second in the three-dimensional space by ab-initio reconstruction with several classes followed by heterogenous refinement. Straight lines indicate the flow of particles and dotted lines the use of particles for the training of a Topaz picking model. Abbreviations: ASU - asymmetric unit, 3DVA - 3D variability analysis.

Methylated ACS binds CO with high affinity (*K*_d_ ≈ 12 nM (ref. ^11^)) to yield an acetyl ligand, indicating almost complete acetylation in the sample used for cryo-EM. After acetylation, most molecules adopt the half-closed state and a minority the wobbly state (Fig. 3 and Extended Data Fig. 5). In the half-closed state, an acetyl moiety is found above Ni_p_, which has a tetrahedral coordination geometry (Fig. 4). This tetrahedral coordination is surprising. In synthetic mimics of cluster A, Ni adopts a square-planar^32^ or trigonal-bipyramidal^33^ conformation after acetylation. Furthermore, previous EXAFS and DFT calculations indicated a square-planar coordination of the acetylated state in monofunctional ACS^16^. Here, the seemingly favourable square-planar coordination with an acetyl ligand is probably prohibited by the clash with Ile149 in the closed state (Supplementary Fig. 16). Previous truncation studies showed that the N-terminal domain plays a critical role in breaking the C–C bond for the reverse reaction of ACS^34^. We propose that this is due to the strain imposed by Ile149 on the geometry of the Ni–acetyl complex in the closed state. However, in the wobbly state, this clash may not be present and Ni_p_ could be square planar, leading to the results observed in the other studies.

Furthermore, in the wobbly state, we observed some density above the N-terminal ACS domain. This density was visible in 2D class averages and an ab initio reconstruction (limited to a resolution of 12 Å), but diminished after refinement to high resolution (Supplementary Fig. 17). This density was also observed more clearly in the CoA-bound state, which is discussed below.

### CoA destabilizes the closed conformation

**Extended Data Fig. 6:**
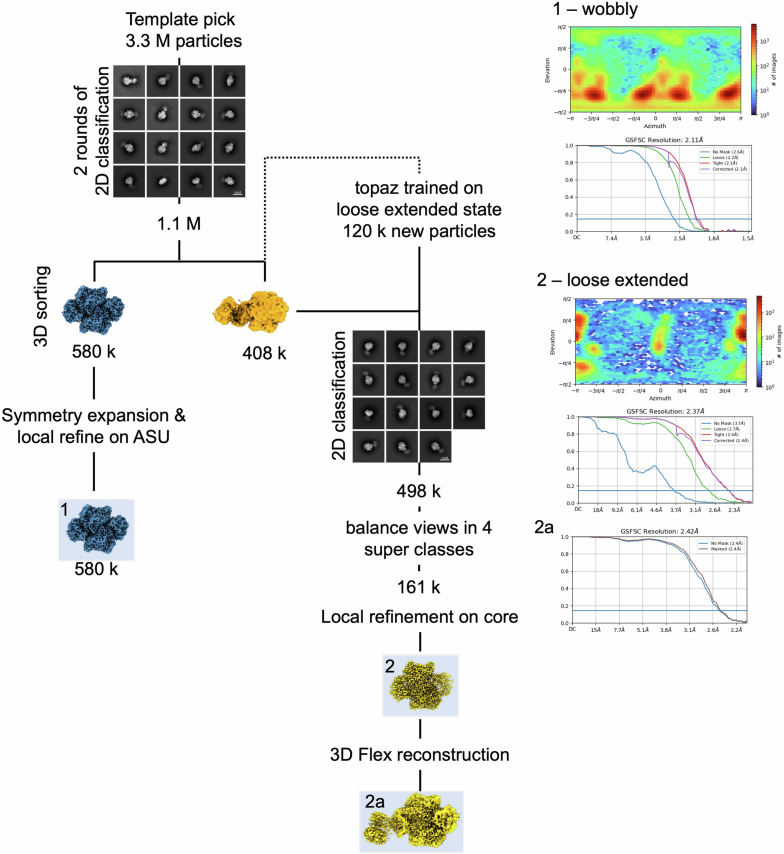
Overview of the particle picking, sorting and 3D reconstruction work flow for the CoA bound data set. Templates for the initial template pick were generated by blob picking the reduced data set followed by 2D classification generating the templates. Template picked particles were sorted in two stages. First, by 2D classification and second in the three-dimensional space by ab-initio reconstruction with several classes followed by heterogenous refinement. Straight lines indicate the flow of particles and dotted lines the use of particles for the training of a Topaz picking model. Abbreviations: ASU - asymmetric unit, 3DVA - 3D variability analysis.

**Extended Data Fig. 7:**
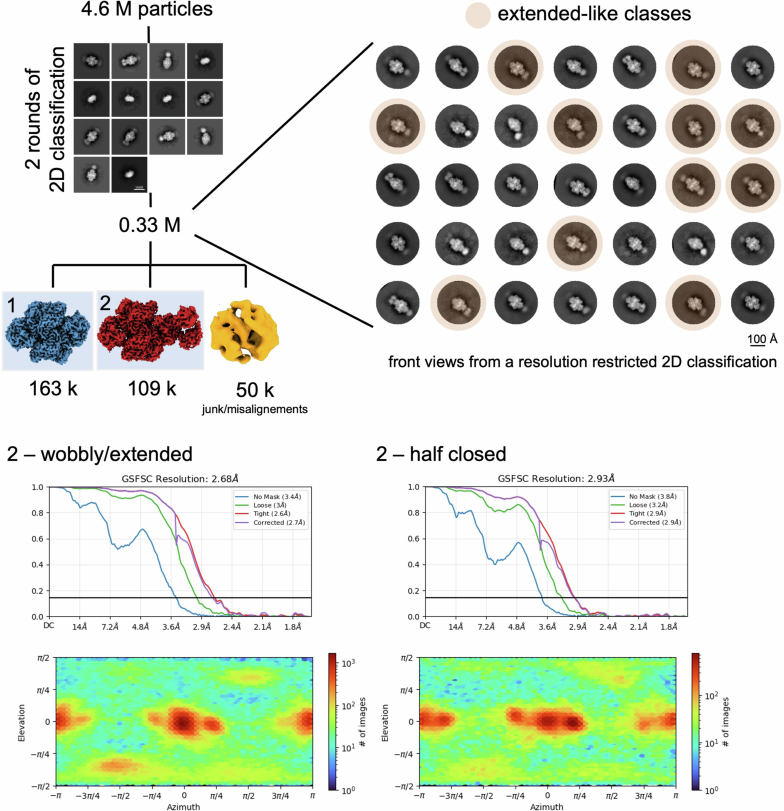
Overview of the particle picking, sorting and 3D reconstruction work flow for the turn over data set. Templates for the initial template pick were generated by blob picking the reduced data set followed by 2D classification generating the templates. Template picked particles were sorted in two stages. First, by 2D classification and second in the three-dimensional space by ab-initio reconstruction with several classes followed by heterogenous refinement. Straight lines indicate the flow of particles. The wobbly state contains a multitude of extended like conformations as can be seen in the aligned 2D class averages showing the front views of CODH–ACS of all selected particles.

The CODH–ACS complex binds CoA with high affinity (*K*_d_ < 88 µM; Supplementary Fig. 4), indicating a saturation of at least 90% under the conditions used for cryo-EM. Isothermal titration calorimetry (ITC) suggests that the binding is slightly driven by entropy, as found for the monomeric ACS subunit^35^. The entropy gain could be due to a major rearrangement of the ACS subunit to a more flexible conformation. In cryo-EM experiments, the wobbly state and a different conformation, which we call the loose extended state, were dominant in the CoA dataset (Fig. 3 and Extended Data Fig. 6). The loose extended conformation seems to have the same domain arrangement as the extended state, but the ACS density is even more blurred than in the extended state, probably due to a greater flexibility of the middle and C-terminal domains (Fig. 5 and Supplementary Fig. 13c,d). Unfortunately, we could not detect CoA in our map. Finally, we wondered how the complex behaves under turnover conditions and collected a dataset at a lower magnification to analyse the species distribution. Upon mixing an acetylated sample with CoA, the percentage of the half-closed conformations dropped from 70% to 40% (Fig. 3). The wobbly state was most abundant and seemed to contain a spectrum of conformations spanning the extended, loose extended and an even more flexible state (Extended Data Fig. 7). We propose that the interaction of CoA with the middle domain, as observed for CODH–ACS of *C. autoethanogenum*^31^, might lead to break up and destabilization of the closed conformation.

## Discussion

We have analysed the structure of the CODH–ACS complex of *C. hydrogenoformans* under six different catalytic conditions using high-resolution cryo-EM. All conditions produced more than one conformation of the CODH–ACS complex. The most populated conformation for all catalytic conditions was the wobbly state, suggesting that this is the energetically favoured state of CODH–ACS. The wobbly state is likely to contain a multitude of ACS conformations. Comparison of the particle distributions showed that 14–32% of the complexes change conformation upon a change of condition, suggesting a sufficient number of active complexes remain despite the expected problems of Zn poisoning of cluster A. We have shown that ligand binding to Ni_p_ at cluster A can be correlated with large conformational changes in the CODH-bound ACS. The gating helix in ACS mediates several conserved interactions between the N-terminal ACS domain and the active site in the C-terminal ACS domain. Our data suggest that steric hindrance between the ligand and gating helix upon ligand binding induces conformational flexibility that prepares the intermediate for the next step in the reaction cycle. Taking all the results together, we obtain four key findings: (1) methylation seems to induce a square-planar coordination and stimulates the closed conformation of ACS, (2) carbonylation favours both the closed and extended conformations, (3) acetylation favours the closed conformation and (4) the presence of CoA strongly disfavours the closed conformation.

**Fig. 6:**
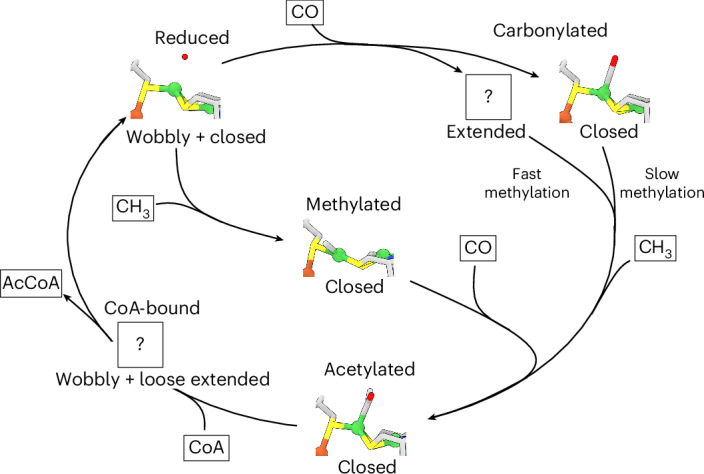
Proposed conformational changes in CODH–ACS and cluster A during catalysis. In the reduced (resting state), CODH–ACS is mainly in the wobbly state. Upon methylation, the closed state of ACS is favoured, in which the tunnel is continuous between cluster A and cluster C. Meanwhile, carbonylation favours the closed and extended state of ACS. The extended state has a methylation-ready conformation and thus probably can be methylated faster than the closed state of ACS, which needs to open to allow the interaction with the corrinoid ring of CoFeSP. At high CO concentrations, the closed conformation might be favoured, resulting in a slowing of the reaction. Upon formation of the Ni–acetyl intermediate, the half-closed state (and the closed state of ACS) is dominant. CoA binding to the reduced state seems to disfavour the closed ACS state and thus can return the protein to the wobbly resting state. The most likely relevant ACS conformation for each step is shown below the structure of Ni_p_. Question marks represent conformations in which cluster A was not resolved at high resolution. The conformations at Ni_p_ are coloured in blue (N), red (O), yellow (S), orange (Fe) and green (Ni).

This led us to propose the conformational cycle of CODH–ACS during catalysis illustrated in Fig. 6. In the reduced state, ACS is prepared to undergo either carbonylation in the closed state, in which the tunnel between cluster A and cluster C is continuous, or methylation by CoFeSP in the wobbly state, in which cluster A can be accessed by solvent. After carbonylation, ACS adopts the extended conformation, in which cluster A can be accessed by CoFeSP and the tunnel is closed, preventing leakage of CO. The closed conformation is present in similar proportions to the extended conformation. However, cluster A in the closed conformation cannot be easily methylated and thus this conformation might be unproductive for catalysis and could be the reason for the observed inhibition of bacterial ACS by CO (refs. ^34,36–38^). Intriguingly, a version of ACS lacking the N-terminal domain, which cannot adopt the closed conformation, is not inhibited by CO (ref. ^34^). In contrast, the closed conformation is productive after methylation of ACS. Under this circumstance, cluster A is ready to accept a CO from the tunnel at the apical position. Accordingly, we observed that almost 50% of the ACS domains in the sample are in the closed conformation in the presence of methyl cobinamide. Compared with the reduced state, Ni_p_ moves in the plane of the ligating cysteines. This is consistent with a square-planar coordination. However, the expected fourth ligand, the methyl moiety, is poorly resolved, which could be due to a clash of the methyl moiety with Ile149, which might introduce strain into the Ni–CH_3_ bond, activating it for condensation with CO. After the formation of the acetylated species, the closed conformation is favoured and Ni_p_ is in a tetrahedral geometry. The acetyl moiety might act as a barb, preventing the gating helix from snapping back to its position in the wobbly state, which also prevents Ni_p_ from attaining a favourable square-planar conformation (as proposed by DFT calculations^16^). This frustration might stabilize the closed conformation. In contrast to the wobbly conformation, the closed conformation shields the highly reactive Ni–acetyl species from the cellular environment so that it cannot be lost by hydrolysis or reactions with other thiols. CoA binding in the final step of the catalytic cycle could force the opening of the closed conformation, allowing thiolysis of the Ni–acetyl bond and release of acetyl-CoA, thereby closing the catalytic cycle. The CODH–ACS complex is an elegant example of how ligand binding orchestrates conformational changes in the complex and vice versa. The protein backbone prevents the loss of valuable intermediates and creates a steric strain in the ligands that activates the Ni–C bonds. While nature has evolved this enzyme for energy efficiency by preventing the loss of CO, that is not crucial for human applications. In a previous study, we showed that CO_2_ reduction is the rate-limiting step of the catalytic process. One way to adapt the enzyme to a higher rate of CO_2_ fixation that emerges from our study would be to destabilize the closed state to prevent self-inhibition by CO and create leaks in the tunnel to increase CO_2_ turnover at cluster C.

## Methods

### Protein purification and sample preparation

CODH–ACS was expressed heterologously in *Escherichia* *coli* M15 and purified as described previously^39^. For the heterologous production of CODH–ACS, *E. coli* M15 pREP4 was transformed with pACSCODH and pKRISC, cultivated in Begg’s medium with FeSO_4_ and l-cysteine at 37 °C under N_2_ bubbling, and induced with isopropyl-β-d-thiogalactoside (IPTG) and NiCl_2_ at an optical density at 600 nm (OD_600_) of 1.6 concomitant with a temperature shift to 42 °C. Cells were collected after 18 h, frozen and stored at −80 °C. CooC1 maturation factor was produced in *E. coli* BL21 (DE3) transformed with pCooC1, grown in Terrific Broth (TB) medium at 37 °C, induced with IPTG at OD_600_ = 0.9 and collected after 3 h. CODH–ACS was purified in an anoxic glove box by resuspending mixed cell pellets in a buffer with sodium dithionite, ATP, MgCl_2_, NiCl_2_, lysozyme and DNase I, followed by sonication and centrifugation. The supernatant was loaded onto a Ni nitrilotriacetate column, eluted with imidazole and subjected to anion exchange chromatography on a Source-30Q column on which ACS and CODH–ACS were separated using a NaCl gradient. CODH–ACS underwent reconstitution with 2-mercaptoethanol and NiCl_2_ at 45 °C for 5 days, followed by size exclusion chromatography on a Superdex S200 column. The purified protein (>95% purity by SDS–PAGE) was concentrated, frozen and stored in liquid N_2_.

CH_3_–CoFeSP was expressed heterologously in *E. coli* BL21 and purified as described by Neumann and Dobek^40^.

AcsC (CfsA) was expressed in *E. coli* Rosetta (DE3) co-transformed with the respective plasmids and pRKISC. Cultures were grown in TB medium with kanamycin, FeSO_4_ and Na_2_S at 37 °C until OD_600_ = 1.2, followed by induction with IPTG after incubation in ice for 20 min. The cultures were supplemented again with FeSO_4_ and Na_2_S 2 h post-induction, and fermentation continued at 25 °C for 22 h before collection. AcsD (CfsB) was expressed in *E. coli* Rosetta (DE3) under similar conditions but without FeSO_4_ and Na_2_S supplementation. For the purification, AcsC and AcsD cell pellets were mixed in a 3:1 ratio, lysed by sonication and cleared by ultracentrifugation. The supernatant was purified by Ni sepharose chromatography, TEV protease cleavage, a second Ni sepharose column separation and Q sepharose chromatography. CoFeSP was incubated with methylcobalamin overnight and further purified via size exclusion chromatography (SEC) on a Superdex 200 column using SEC buffer. The final protein was concentrated to 20–25 mg ml^−1^, frozen and stored in liquid nitrogen. Samples were prepared in an anoxic box filled with N_2_. CODH–ACS was diluted in 50 mM MOPS–NaOH (pH 7.6) with 150 mM NaCl to 0.565 g l^−1^. The different states of the complex were trapped by adding different supplements and incubation at 22 °C (Extended Data Table 1). All mixtures were prepared in 1.5 ml closed reagent tubes containing 100 µl of sample. The number of generated CO molecules for the acetylated and carbonylated samples was calculated using equation (1) based on the measured maximum velocity *V*_max_ for CO_2_ reduction and our previous kinetic characterization of CODH–ACS:

1 $$V\approx {V}_{\rm{max }} \times F_{{\rm{temperature}}}\times {F}_{{\rm{reagents}}}$$

where *V* is the estimated activity, *V*_max_ ≈ 8 min^−1^, *F*_temperature_ is the factor accounting for the lower temperature at sample preparation (20 °C instead of 50 °C) and *F*_reagents_ is the factor accounting for the lower concentrations of substrates due to the needs of cryo-EM. Based on our previous results, *F*_temperature_ ≈ 0.1 (based on the Arrhenius plot)^39^ and *F*_reagents_ ≈ 0.8 (based on the activity-vs-substrate concentration (*V*–*S*) characteristics). Thus, we have an activity of ~0.064 min^−1^, leading to the generation of ~60 CO molecules per CODH within 16 h.

We purified two batches of CODH–ACS. One batch was used for Cryo-EM experiments and activity measurements, and the other batch was used for ITC and UV–Vis experiments.

### Cryo-EM grid preparation

Grids were plunge-frozen in an anoxic box filled with N_2_ using a custom-made manual plunger (Neptune Fluid Flow Systems). UltraAufoil 1.2/1.3 300 grids (Quantifoil) were glow-discharged for 30 s at a vacuum pressure of <0.2 mbar and a flux of 304 mA. Then, 3.5 µl of the CODH–ACS sample (565 µg ml^−1^) was suspended on the freshly glow-discharged grids and incubated for 45 s. Excess solution was removed by blotting with filter paper (Whatman) for 5 s and the grid was immediately plunged into liquid ethane. The relative humidity inside the box was maintained between 70% and 80% and the temperature between 20 and 16 °C.

### Cryo-EM data collection

Cryo-EM movies were recorded at CEITEC (Brno, Czech Republic) using the aberration-free image shift on a Titan Krios device equipped with a Bioquantum K3 camera and an energy filter (slit width 10 eV) with SerialEM 4.1 (ref. ^41^). The pixel size was 0.5113 Å and movies were recorded with 40 frames with a total dose of ~50 e^−^ Å^−^^2^. The set defocus range was −2.8 to −0.8 µm. The turnover dataset was collected with a pixel size of 0.8336 Å. The CODH–ACS + CoFeSP dataset was collected using our in-house microscope (Talos F200) at 200 kV and a FalconIII camera with 33 frames. An overview of the data sets is given in Extended Data Table 2.

### Movie processing and contrast transfer function (CTF) determination

Movies were aligned using Cryosparcs (v4.3.0)^42^ built-in Patch Motion Correction program. The defocus and astigmatism were determined using the built-in Patch CTF program. Micrographs were discarded if the astigmatism was higher than 2,000 Å or if the CTF could only be fitted to a resolution of less than 4 Å.

### Particle picking and curation

Initial particle picking was performed with 2D class averages from the reduced dataset as templates. Particles were sorted in two rounds of 2D classification followed by 3D classification with typically four classes using ab initio reconstruction followed by heterogeneous refinement. For 2D and 3D classification, only those classes showing any hint of a CODH core were kept. Less than 100,000 particles were obtained for some species. Then we used the Topaz deep learning picker to detect more of these particles. The particle curation workflows are described in more detail for all datasets in Extended Data Figs. 1–7.

### Three-dimensional reconstruction

For the final reconstruction, particles were extracted in boxes of 720 pixels (~370 Å). These were down-sampled to 360 pixels, or 512 pixels if the Nyquist limit was reached. Maps were reconstructed with non-uniform refinement implemented in Cryosparc with the fitting of higher order aberrations and per-particle-defocus enabled. Where possible, the dataset was split into 45 exposure groups based on similar beam shifts at acquisition. Species showing *C*_2_ symmetry (wobbly and closed) were first refined in *C*_2_ and the particle set was then symmetry-expanded. These symmetry-expanded particles were used for local refinement on one asymmetric unit (CODH + N-terminal ACS domain (wobbly) or CODH + ACS (closed)). This procedure did not yield a gain in resolution but allowed focused classification on symmetry-related active site clusters.

### Focused classification on the active site clusters

The classification on cluster C was carried out for the wobbly species as it yielded the highest resolution. After generating a mask extending 12 Å from cluster C and a soft drop-off for 5 Å, 3D variability analysis^43^ was carried out with a band pass filter from 2.2 to 10 Å in three modes. The dataset was then split into three clusters using 3D variability display, for which the density at cluster C was examined. The classification on cluster A was carried out in the same way, but on the half-closed and closed species.

### Three-dimensional flexible refinement

Reconstruction of a consensus map for the extended state and its deformation were calculated using 3DFlex (ref. ^44^) using the default parameters. Only the latent centring strength and rigidity prior during learning were adjusted so that the particles populated the latent space between 1.5 and −1.5. The number of latent dimensions were selected on the basis of the loss of the training process.

### Cryo-EM model building and analysis

Atomic models were constructed using the X-ray crystal structure of CODH–ACS as a template (PDB no. 7ZKJ). Water atoms were built in the model using phenix.douse. The template model including the specific ligand was refined with phenix.refine^45^ for 16 cycles with individual atomic displacement parameters (ADP), occupancy, Asn-Gln-His (NQH)-flip and global minimization options turned on and a target RMSD of 0.01 Å for bonds and 0.1° for angles. Afterwards the difference map calculated with Servalcat^46^ was manually inspected in Coot^47^ and the model adjusted if necessary. This adjustment was followed by six cycles of phenix.refine. Metal–sulfur bonds in the clusters were restrained to the bond lengths found in crystal structures with a standard deviation of 0.1 Å. The Ni–CH_3_, Ni–CO and Ni–acetyl bond lengths were determined to be 1.98 ± 0.1,1.75 ± 0.2 and 1.9 ± 0.2 Å, respectively. Furthermore, the CO and acetyl moiety were restrained to be in-plane with the Ni–C bond. The extended state was modelled using the AlphaFold3 model^28^ calculated for the ACS–CoFeSP complex combined with the wobbly CODH–ACS model. All structures were visualized using ChimeraX 1.7 (ref. ^48^). Tunnels were calculated using Caveranalyst 2.0 (ref. ^11^) using the default settings.

### Sequence analysis

The ACS sequence was used as a query for PSI-BLAST (ref. ^49^) against the non-redundant protein sequences database using the National Center for Biotechnology Information (NCBI) web server with the default parameters (https://blast.ncbi.nlm.nih.gov/Blast.cgi). The hit sequences were aligned using COBALT (ref. ^50^) on the NCBI website. PSI-BLAST was used to obtain a diverse distribution of hits. To further diversify the found sequences, only hits with a sequence identity of less than 98% were kept, resulting in 334 sequences. These were analysed with the Biopython package^51^ and Jalview^52^, and a tree based on the sequence identity distance was built using the tree tool from the Biopython package.

### ITC experiments

ITC data were collected using a MicroCal VP-ITC instrument (Malvern Panalytical) inside an anoxic glove box at 25 °C in a buffer solution comprising 50 mM MOPS (pH 7.6) and 150 mM NaCl supplemented with 1 mM Ti(III)–EDTA. Samples were incubated in the respective assay buffer for 2 h at 22 °C before the titration experiments. For the CoA titrations, the cell contained 20 µM CODH–ACS (*V* = 1.4 ml) and the syringe 596 µM CoA, and for the methyl cobinamide titrations, the cell contained 20 µM CODH–ACS and the syringe 476 µM methyl cobinamide. The reference power was 18 µcal s^−1^ and the stirring speed was 307 r.p.m. Raw thermograms were baseline-corrected and integrated using NITPIC (ref. ^53^) and binding isotherms were fitted using SEDPHAT (ref. ^54^). The titrations of methyl cobinamide were further analysed with Dynafit4 (ref. ^55^) because, to our knowledge, no other software has the possibility to fit a two-substrate-two-product mechanism. The ITC data were prepared for Dynafit4 as follows using the Python script detailed in Supplementary Note 2: (1) the fraction of competent ACS was determined with SEDPHAT and (2) the ACS concentration was corrected accordingly and the differential heat of each injection was cumulatively added. These data were then analysed with Dynafit4 using the script shown in Supplementary Note 3.

### UV–Vis spectroscopy

The reduction of CODH–ACS with Ti(III)–EDTA was followed using an Agilent 8453 UV–Vis spectrophotometer at 25 °C. CODH–ACS (14.7 µM) in MOPS–NaOH (pH 7.6) with 150 mM NaCl was rapidly mixed with Ti(III)–EDTA (final concentration 1 mM) and the change in absorbance was followed for 5 h.

### Activity measurements

CO oxidation was measured in sealed cuvettes at 70 °C in 50 mM HEPES (pH 8), 2 mM dithiothreitol (DTT) and 20 mM methyl viologen buffer saturated with CO by following the increase in absorbance at 440 nm due to the reduction of methyl viologen, as described in Svetlitchnyi et al.^13^. CODH–ACS was pre-incubated for 1 h at 25 °C in 50 mM HEPES (pH 8), 2 mM DTT and 1 mM dithionite to ensure its full reduction. CO_2_ reduction was measured by the change in absorbance of haemoglobin at 420 and 431 nm upon binding CO, as described by Ruickoldt et al.^20^. The assay was performed in sealed cuvettes in 100 mM MOPS (pH 7.2) buffer supplemented with 3 mg l^−1^ carbonic anhydrase and 1.19 mM CO_2_, 20.9 mM Ti(III)–EDTA and 7 µM haemoglobin. Acetyl-CoA was synthesized from CO_2_ as described by Ruickoldt et al.^11^ at 50 °C in sealed cuvettes. The reaction was followed at 387 nm, which is indicative of the transition of methyl cobinamide to cobinamide. The assay buffer comprised 100 mM MOPS (pH 7.2) supplemented with 3 mg l^−1^ carbonic anhydrase and 1.15 mM CO_2_, 10 mM Ti(III)–EDTA and 50 µM methyl cobinamide. The reaction was started by adding 200 µM CoA.

### Metal content analysis by total reflection X-ray fluorescence spectroscopy

The metal content present in the CODH–ACS complex was quantitatively determined by total reflection X-ray fluorescence spectroscopy (TXRF) using a Bruker S4 T-Star spectrometer (Bruker Nano) with Mo Kα monochromatization. Samples were excited for 300 s and an X-flash silicon drift detector was used for X-ray acquisition. Samples were diluted 1:1 with a 100 mg l^−1^ Certipur yttrium metal standard (Merck) and 4 µl aliquots were adhered onto siliconized quartz glass carriers (Bruker Nano) by drying on a hot plate at 50 °C. To assess the metal content in the buffer, identical samples containing buffer without the CODH–ACS complex were measured in parallel. The amount of metal in the samples was determined relative to the 50 mg l^−1^ yttrium reference sample and calculated as micromolar quantities. Four technical replicates were measured per sample. Analytical spectral analysis and deconvolution were performed using Esprit 1.0 (Bruker Nano).

### Reporting summary

Further information on research design is available in the Nature Portfolio Reporting Summary linked to this article.

## Supplementary information

Supplementary Information Supplementary Discussion, Figs. 1–17 and Table 1.

Reporting Summary

Supplementary Video 1 Movements in the latent space deduced with 3D flexible refinement for the loose extended species in the dataset in the presence of CoA and the extended species in the carbonylated dataset.

## Figures and Tables

**Extended Data Table 1:**
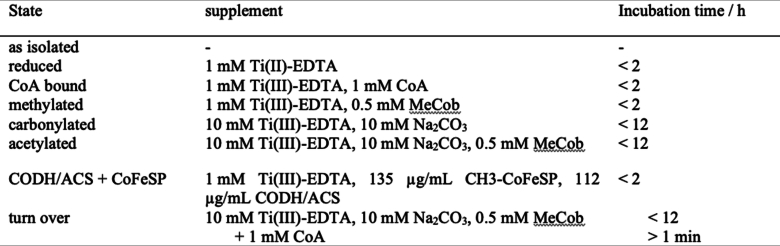
Composition of the different samples

**Extended Data Table 2:**
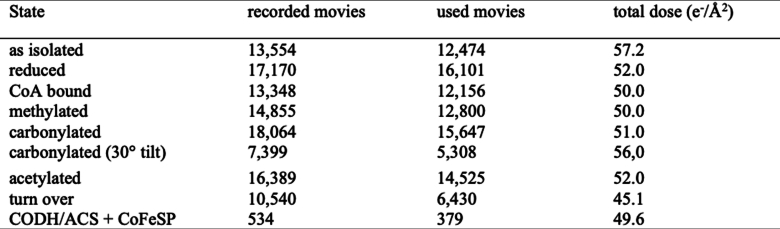
Overview of the collected data sets

**Extended Data Table 3:**
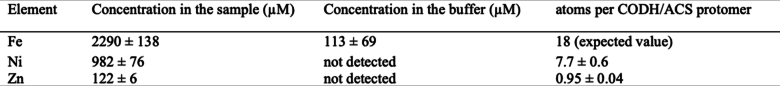
Results of the TXRF metal content analysis

Shown is the mean of four technical replicates and its standard deviation. CODH–ACS was used at a concentration of 63.7 µM. The atoms per CODH–ACS were calculated based on the Fe concentration asssuming 18 Fe per CODH–ACS protomer.

## Data Availability

All data are available from the authors upon reasonable request. The maps (and structures) have been deposited in the PDB and Electron Microscopy Data Bank under the following accession codes: EMD-50837, EMD-50729, 9FU3/EMD-50754, 9FUC/EMD-50761, 9FR0/EMD-50674, 9FU4/EMD-50756, 9FU7/EMD-50757, 9FU9/EMD-50758, 9FUA/EMD-50759, 9FUB/EMD-50760, 9FR1/EMD-50677, 9FOX/EMD-50631, 9FOP/EMD-50626, 9FO4/EMD-50616, 9FNJ/EMD-50598 and 9FNC/EMD-50588.

## Code availability

The scripts used for data analysis are available in the Supplementary Information.

## References

[1] Martin, W. F. Older than genes: the acetyl CoA pathway and origins. *Front. Microbiol.* **11**, 817 (2020). 32655499 10.3389/fmicb.2020.00817 PMC7325901

[2] Martin, W. F. Carbon–metal bonds: rare and primordial in metabolism. *Trends Biochem. Sci.* **44**, 807–818 (2019). 31104860 10.1016/j.tibs.2019.04.010

[3] Russell, M. J. & Martin, W. The rocky roots of the acetyl-CoA pathway. *Trends Biochem. Sci.* **29**, 358–363 (2004). 15236743 10.1016/j.tibs.2004.05.007

[4] Ragsdale, S. W. & Wood, H. G. Acetate biosynthesis by acetogenic bacteria. *J. Biol. Chem.* **260**, 3970–3977 (1985). 2984190

[5] Ljungdahl, L. G. Acetate synthesis in aceto genic bacteria. *Annu. Rev. Microbiol.* **40**, 415–450 (1986). 3096193 10.1146/annurev.mi.40.100186.002215

[6] Fackler, N. et al. Stepping on the gas to a circular economy: accelerating development of carbon-negative chemical production from gas fermentation. *Annu. Rev. Chem. Biomol. Eng.* **12**, 439–470 (2021). 33872517 10.1146/annurev-chembioeng-120120-021122

[7] Ragsdale, S. W. Enzymology of the Wood–Ljungdahl pathway of acetogenesis. *Ann. N. Y. Acad. Sci.* **1125**, 129–136 (2008). 18378591 10.1196/annals.1419.015 PMC3040112

[8] Doukov, T. I., Iverson, T. M., Seravalli, J., Ragsdale, S. W. & Drennan, C. L. A Ni-Fe-Cu center in a bifunctional carbon monoxide dehydrogenase/acetyl-CoA synthase. *Science* **298**, 567–572 (2002). 12386327 10.1126/science.1075843

[9] Darnault, C. et al. Ni-Zn-[Fe_4_-S_4_] and Ni-Ni-[Fe_4_-S_4_] clusters in closed and open α subunits of acetyl-CoA synthase/carbon monoxide dehydrogenase. *Nat. Struct. Biol.* **10**, 271–279 (2003). 12627225 10.1038/nsb912

[10] Doukov, T. I., Blasiak, L. C., Seravalli, J., Ragsdale, S. W. & Drennan, C. L. Xenon in and at the end of the tunnel of bifunctional carbon monoxide dehydrogenase/acetyl-CoA synthase. *Biochemistry* **47**, 3474–3483 (2008). 18293927 10.1021/bi702386t PMC3040099

[11] Ruickoldt, J. et al. Coupling CO_2_ reduction and acetyl‐CoA formation: the role of a CO capturing tunnel in enzymatic catalysis. *Angew. Chem. Int. Ed.* **63**, e202405120 (2024). 10.1002/anie.202405120 38743001

[12] Lemaire, O. N. & Wagner, T. Gas channel rerouting in a primordial enzyme: structural insights of the carbon-monoxide dehydrogenase/acetyl-CoA synthase complex from the acetogen *Clostridium autoethanogenum*. *Biochim. Biophys. Acta Bioenerg.* **1862**, 148330 (2021). 33080205 10.1016/j.bbabio.2020.148330

[13] Svetlitchnyi, V. et al. A functional Ni-Ni-[4Fe-4S] cluster in the monomeric acetyl-CoA synthase from *Carboxydothermus hydrogenoformans*. *Proc. Natl Acad. Sci. USA* **101**, 446–451 (2004). 14699043 10.1073/pnas.0304262101 PMC327167

[14] Cohen, S. E. et al. Negative-stain electron microscopy reveals dramatic structural rearrangements in Ni-Fe-S-dependent carbon monoxide dehydrogenase/acetyl-CoA synthase. *Structure* **29**, 43–49.e3 (2021). 32937101 10.1016/j.str.2020.08.011 PMC7796957

[15] Seravalli, J. & Ragsdale, S. W. Pulse-chase studies of the synthesis of acetyl-CoA by carbon monoxide dehydrogenase/acetyl-CoA synthase: evidence for a random mechanism of methyl and carbonyl addition. *J. Biol. Chem.* **283**, 8384–8394 (2008). 18203715 10.1074/jbc.M709470200 PMC2820341

[16] Can, M. et al. Characterization of methyl- and acetyl-Ni intermediates in acetyl CoA synthase formed during anaerobic CO_2_ and CO fixation. *J. Am. Chem. Soc.* **145**, 13696–13708 (2023). 37306669 10.1021/jacs.3c01772 PMC10311460

[17] Schrapers, P. et al. Ligand binding at the A-cluster in full-length or truncated acetyl-CoA synthase studied by X-ray absorption spectroscopy. *PLoS ONE* **12**, e0171039 (2017). 28178309 10.1371/journal.pone.0171039 PMC5298270

[18] Bramlett, M. R. et al. Mössbauer and EPR study of recombinant acetyl-CoA synthase from *Moorella thermoacetica*. *Biochemistry* **45**, 8674–8685 (2006). 16834342 10.1021/bi060003+

[19] Cohen, S. E. et al. Crystallographic characterization of the carbonylated A-cluster in carbon monoxide dehydrogenase/acetyl-CoA synthase. *ACS Catal.* **10**, 9741–9746 (2020). 33495716 10.1021/acscatal.0c03033 PMC7819276

[20] Ruickoldt, J., Basak, Y., Domnik, L., Jeoung, J.-H. & Dobbek, H. On the kinetics of CO_2_ reduction by Ni,Fe-CO dehydrogenases. *ACS Catal.* **12**, 13131–13142 (2022).

[21] Mayhew, S. G. The redox potential of dithionite and SO^−^_2_ from equilibrium reactions with flavodoxins, methyl viologen and hydrogen plus hydrogenase. *Eur. J. Biochem.* **85**, 535–547 (1978). 648533 10.1111/j.1432-1033.1978.tb12269.x

[22] Feng, J. & Lindahl, P. A. Carbon monoxide dehydrogenase from *Rhodospirillum rubrum*: effect of redox potential on catalysis. *Biochemistry* **43**, 1552–1559 (2004). 14769031 10.1021/bi0357199

[23] Can, M., Armstrong, F. A. & Ragsdale, S. W. Structure, function, and mechanism of the nickel metalloenzymes, CO dehydrogenase, and acetyl-CoA synthase. *Chem. Rev.* **114**, 4149–4174 (2014). 24521136 10.1021/cr400461p PMC4002135

[24] Burton, R., Can, M., Esckilsen, D., Wiley, S. & Ragsdale, S. W. in *Methods in Enzymology* Vol. 613: Enzymes of Energy Technology (ed Armstrong, F.) Ch. 12 (Elsevier, 2018). 10.1016/bs.mie.2018.10.005 PMC6309614 30509471

[25] Elghobashi-Meinhardt, N., Tombolelli, D. & Mroginski, M. A. QM/MM computations reveal details of the acetyl-CoA synthase catalytic center. *Biochim. Biophys. Acta Gen. Subj.* **1864**, 129579 (2020). 32135171 10.1016/j.bbagen.2020.129579

[26] Can, M., Giles, L. J., Ragsdale, S. W. & Sarangi, R. X-ray absorption spectroscopy reveals an organometallic Ni–C bond in the CO-treated form of acetyl-CoA synthase. *Biochemistry* **56**, 1248–1260 (2017). 28186407 10.1021/acs.biochem.6b00983 PMC5710745

[27] Kung, Y. et al. Visualizing molecular juggling within a B12-dependent methyltransferase complex. *Nature* **484**, 265–269 (2012). 22419154 10.1038/nature10916 PMC3326194

[28] Abramson, J. et al. Accurate structure prediction of biomolecular interactions with AlphaFold 3. *Nature* **630**, 493–500 (2024). 38718835 10.1038/s41586-024-07487-w PMC11168924

[29] Svetlitchnaia, T., Svetlitchnyi, V., Meyer, O. & Dobbek, H. Structural insights into methyltransfer reactions of a corrinoid iron–sulfur protein involved in acetyl-CoA synthesis. *Proc. Natl Acad. Sci. USA* **103**, 14331–14336 (2006). 16983091 10.1073/pnas.0601420103 PMC1599964

[30] Hennig, S. E. et al. ATP-induced electron transfer by redox-selective partner recognition. *Nat. Commun.* **5**, 4626 (2014). 25109607 10.1038/ncomms5626

[31] Yin, M. D., Lemaire, O. N., Guadalupe, J., Jiménez, R. & Belhamri, M. Conformational dynamics of a multienzyme complex in anaerobic carbon fixation. *Science* **387**, 498–504 (2025). 10.1126/science.adr9672 39883773

[32] Stavropoulos, P., Muetterties, M. C., Carriè, M. & Holm, R. H. Structural and reaction chemistry of nickel complexes in relation to carbon monoxide dehydrogenase: a reaction system simulating acetyl-coenzyme A synthase activity. *J. Am. Chem. Soc.* **113**, 8485–8492 (1991).

[33] Ito, M., Kotera, M., Matsumoto, T. & Tatsumi, K. Dinuclear nickel complexes modeling the structure and function of the acetyl CoA synthase active site. *Proc. Natl Acad. Sci. USA* **106**, 11862–11866 (2009). 19584250 10.1073/pnas.0900433106 PMC2715534

[34] Gencic, S., Duin, E. C. & Grahame, D. A. Tight coupling of partial reactions in the acetyl-CoA decarbonylase/synthase (ACDS) multienzyme complex from *Methanosarcina thermophila*: acetyl C–C bond fragmentation at the A cluster promoted by protein conformational changes. *J. Biol. Chem.* **285**, 15450–15463 (2010). 20202935 10.1074/jbc.M109.080994 PMC2865265

[35] Kreibich, J. *Mechanistic Insights into Carbon Monoxide and CoA Binding at the Ni,Ni-[4Fe-4S] Active Site of the Acetyl-CoA Synthase from* Carboxydothermus hydrogenoformans. PhD thesis, Humboldt Univ. Berlin (2021).

[36] Gencic, S., Kelly, K., Ghebreamlak, S., Duin, E. C. & Grahame, D. A. Different modes of carbon monoxide binding to acetyl-CoA synthase and the role of a conserved phenylalanine in the coordination environment of nickel. *Biochemistry* **52**, 1705–1716 (2013). 23394607 10.1021/bi3016718

[37] Maynard, E. L. & Lindahl, P. A. Catalytic coupling of the active sites in acetyl-CoA synthase, a bifunctional CO-channeling enzyme. *Biochemistry* **40**, 13262–13267 (2001). 11683635 10.1021/bi015604+

[38] Maynard, E. L., Sewell, C. & Lindahl, P. A. Kinetic mechanism of acetyl-CoA synthase: steady-state synthesis at variable CO/CO_2_ pressures. *J. Am. Chem. Soc.* **123**, 4697–4703 (2001). 11457278 10.1021/ja004017t

[39] Ruickoldt, J. *On the Coupling of the Catalytical Activities of the CODH/ACS Complex from* Carboxydothermus hydrogenoformans. PhD thesis, Humboldt Univ. Berlin (2022).

[40] Neumann, F. & Dobbek, H. ATP binding and a second reduction enables a conformationally gated uphill electron transfer. *ACS Catal.* **11**, 8565–8575 (2021).

[41] Mastronarde, D. N. SerialEM: a program for automated tilt series acquisition on Tecnai microscopes using prediction of specimen position. *Microsc. Microanal.* **9**, 1182–1183 (2003).

[42] Punjani, A., Rubinstein, J. L., Fleet, D. J. & Brubaker, M. A. CryoSPARC: algorithms for rapid unsupervised cryo-EM structure determination. *Nat. Methods* **14**, 290–296 (2017). 28165473 10.1038/nmeth.4169

[43] Punjani, A. & Fleet, D. J. 3D variability analysis: resolving continuous flexibility and discrete heterogeneity from single particle cryo-EM. *J. Struct. Biol.* **213**, 107702 (2021). 33582281 10.1016/j.jsb.2021.107702

[44] Punjani, A. & Fleet, D. J. 3DFlex: determining structure and motion of flexible proteins from cryo-EM. *Nat. Methods* **20**, 860–870 (2023). 37169929 10.1038/s41592-023-01853-8 PMC10250194

[45] Liebschner, D. et al. Macromolecular structure determination using X-rays, neutrons and electrons: recent developments in *Phenix*. *Acta Crystallogr. D* **75**, 861–877 (2019). 10.1107/S2059798319011471 PMC6778852 31588918

[46] Yamashita, K., Palmer, C. M., Burnley, T. & Murshudov, G. N. Cryo-EM single-particle structure refinement and map calculation using Servalcat. *Acta Crystallogr. D* **77**, 1282–1291 (2021). 10.1107/S2059798321009475 PMC8489229 34605431

[47] Emsley, P., Lohkamp, B., Scott, W. G. & Cowtan, K. Features and development of *Coot*. *Acta Crystallogr. D* **66**, 486–501 (2010). 20383002 10.1107/S0907444910007493 PMC2852313

[48] Pettersen, E. F. et al. UCSF ChimeraX: structure visualization for researchers, educators, and developers. *Protein Sci.* **30**, 70–82 (2021). 32881101 10.1002/pro.3943 PMC7737788

[49] Altschul, S. F. et al. Gapped BLAST and PSI-BLAST: a new generation of protein database search programs. *Nucleic Acids Res.* **25**, 3389–3402 (1997). 9254694 10.1093/nar/25.17.3389 PMC146917

[50] Papadopoulos, J. S. & Agarwala, R. COBALT: constraint-based alignment tool for multiple protein sequences. *Bioinformatics* **23**, 1073–1079 (2007). 17332019 10.1093/bioinformatics/btm076

[51] Cock, P. J. A. et al. Biopython: freely available Python tools for computational molecular biology and bioinformatics. *Bioinformatics* **25**, 1422–1423 (2009). 19304878 10.1093/bioinformatics/btp163 PMC2682512

[52] Waterhouse, A. M., Procter, J. B., Martin, D. M. A., Clamp, M. & Barton, G. J. Jalview Version 2—a multiple sequence alignment editor and analysis workbench. *Bioinformatics* **25**, 1189–1191 (2009). 19151095 10.1093/bioinformatics/btp033 PMC2672624

[53] Keller, S. et al. High-precision isothermal titration calorimetry with automated peak-shape analysis. *Anal. Chem.* **84**, 5066–5073 (2012). 22530732 10.1021/ac3007522 PMC3389189

[54] Zhao, H., Piszczek, G. & Schuck, P. SEDPHAT—a platform for global ITC analysis and global multi-method analysis of molecular interactions. *Methods* **76**, 137–148 (2015). 25477226 10.1016/j.ymeth.2014.11.012 PMC4380758

[55] Kuzmič, P. DynaFit—a software package for enzymology. *Methods Enzymol.* **467**, 247–280 (2009). 19897096 10.1016/S0076-6879(09)67010-5

