# Supplementary material for: Ligand binding to a Ni–Fe cluster orchestrates conformational changes of the CO-dehydrogenase–acetyl-CoA synthase complex
Source: Nat Catal. 2025 Jul 11;8(7):657–67. doi: 10.1038/s41929-025-01365-y (PMC12289521; doi:10.1038/s41929-025-01365-y)
Supplement: Supplementary file 1 — Supplementary Discussion, Figs. 1–17 and Table 1. [file 41929_2025_1365_MOESM1_ESM.pdf]

nature catalysis

Supplementary information https://doi.org/10.1038/s41929-025-01365-y

Ligand binding to a Ni–Fe cluster orchestrates conformational changes of the CO-dehydrogenase–acetyl-CoA synthase complex

In the format provided by the authors and unedited

Supplementary Information

Supplementary Discussion

Analysis of the triangle species in the as isolated data set

In the triangle species, three ACS-MC domains assemble to an equilateral trimer, while the CODH and the ACS N-terminal domains are only visible in the 2D-class averages as smeared densities (Supplementary Figure 1a). A set of 104.000 particles was used for the best isotropic map resolved to 3.42 Å, showing that the trimer is stabilized by the interaction of the C-terminal 6x-His tag of one ACS with the proximal Ni of another one (Supplementary Data Figure 2b). The triangle state is not physiologically relevant and does not seem to cause any of the other observed conformations of the CODH/ACS complex: Only 22 % or 27.8 % of the particles classified as wobbly or half-closed were in interaction distance to any of the ~250.000 particles classified as triangles (Supplementary Figure 1c).

Analysis of the effect of Zn poisoning on the data set

It is long known that Zn ions readily replace the proximal Ni in cluster A. Although we took utmost care to prevent this poisoning by washing all glass ware with HCl and by treating all buffers with the metal-absorbing resin Chelex 100, cluster A was still poisoned by Zn. In a previous study, we have purified several variants of CODH/ACS and estimated the Ni content at the proximal site by anomalous scattering to be 38±9 % (n = 5). The other ~60% of ACS contained probably Zn. Zn containing ACS is inactive, Zn is tetraedrally coordinated and binds a water or other solvent-derived ligand (acetate, Cl^-, I^- or similar). Thus, it looks like the cluster A found in the as-isolated and reduced state. However, Ni can also adopt a tetrahedral conformation and bind those ligands. Acetylation, carbonylation and methylation is not possible on Zn. Furthermore, Zn cannot attain a square-planar conformation. Thus, any observed change at cluster A towards a square-planar coordination cannot be biased or flawed by Zn poisoning.

Regarding the conformation of Zn containing ACS little is known. Zn containing ACS can adopt the closed state as it crystallizes in that form. However, its conformation should not be influenced by the substrates as it cannot be methylated, carbonylated nor acetylated. Probably, it is also able to adopt the wobbly state as this state is the only invariant and common state in all data sets. In the reduced and as-isolated state Zn containing ACS is probably distributed between the wobbly and half-closed state. In the worst case, all half-closed ACS in that data set contain Zn and the Ni-containing ones are wobbly. Then across all data sets one would expect a constant population of 15% half-closed ACS with Zn. Even in that case, the closed states of ACS observed upon acetylation and methylation would be mainly occupied with Ni as 35% and 47% of all ACS in theses data sets have the closed conformation.

Supplementary Table S1: Refinement and model statistics.

Data set	As isolated			Reduced		Acetylated		Carbonylated		Methylated				CoA
**State**	Half-closed	wobbly	triangle	Half-closed	wobbly	Half-closed	Wobbly	Half-closed	wobbly	Half-closed (Class 2)	Half-closed (Class 1)	Closed (Class 1)	Wobbly	Wobbly
**Resolution**	2.2	1.9	3.42	2.3	2.04	2.0	2.05	2.21	1.98	2.47	2.33	2.35	2.24	2.06
**CC mask**	0.799	0.859	0.644	0.816	0.864	0.866	0.87	0.83	0.87	0.883	0.888	0.807	0.881	0.855
**Clashscore**	5.2	3.14	19.4	5.8	2.95	3.66	3.28	4.98	2.49	3.72	3.18	5.91	3.08	3.47
**build atoms**	18793	7973	10123	18817	8090	18927	8152	18850	8131	18822	18927	11224	8218	8176
**build waters**	238	356	126	262	473	349	535	259	514	266	372	286	601	559
**Ramachandran statistics**														
**outlier**	0.13	0.1	0.4	0.08	0.0	0.08	0.1	0.17	0.0	0.08	0.04	0.07	0.0	0.0
**allowed**	3.16	2.97	8.23	3.16	3.28	2.91	2.15	2.95	2.97	3.16	3.04	3.01	3.18	2.87
**favored**	96.7	96.9	91.3	96.7	96.7	97.0	97.7	96.8	97.0	96.7	96.9	96.9	96.8	97.1
**RMSD**														
**bond (Å)**	0.003	0.003	0.019	0.003	0.004	0.003	0.002	0.003	0.004	0.003	0.003	0.004	0.003	0.003
**angle (°)**	0.527	0.587	0.659	0.54	0.633	0.569	0.495	0.541	0.665	0.493	0.55	0.59	0.575	0.591
**mean B-factor protein**	54.44	9.44	50.48	39.05	4.97	28.01	24.04	48.39	17.53	42.49	36.78	36.17	26.0	18.95
**mean B-factor waters**	54.33	9.61	43.82	39.09	5.19	30.13	24.54	45.25	24.88	41.99	39.22	27.95	35.25	26.19

**C600-Nip-C512 angle (°)**	131.09	-		121.31	-	135.36	-	128.36	-	151.0	145.55	147.78	-	-
**Cluster C Ni occupancy**	-	0.11		-	0.15	-	0.12		0.12				0.05	0.0
**B-factor used for sharpening (Å^2)**	-37.2	-46.9	-108.1	-55.0	-58.5	-46.2	-42.8	-42.9	-47.4	-62.9	-68.2	-66.3	-71.0	-65.2

```
import paramiko
from paramiko.client import SSHClient, AutoAddPolicy
import pandas as pd
from cryosparc.dataset import Dataset
import numpy as np
from matplotlib import pyplot as plt

#This code block defines the function to create a coordinate list from the ..._passthroug_particles.cs file. Afterwards, the coordinate lists of two data sets are created

def create_coordinates_list(cs_file="xxx.cs"):
  sftp_client = client.open_sftp()
  with sftp_client.open(cs_file) as f:
    f.prefetch()
    cs_dict = Dataset.load(f)
  client.close


  keys = ['location/micrograph_uid','location/center_x_frac','location/center_y_frac']
  particle_dict = {x:cs_dict[x] for x in keys}
  particles = pd.DataFrame.from_dict(particle_dict)


  particles['x'] = particles["location/center_x_frac"] * 5760 *0.5113
  particles['y'] = particles["location/center_y_frac"] * 4092 *0.5113

  display(particles)
  return particles

J80 = create_coordinates_list(cs_file="/path/to/J80_passthrough_particles.cs")
J101 = create_coordinates_list(cs_file="/path/to/J101_passthrough_particles_all_classes.cs")

## Calculating the distances

#First, a function for distance calculation is defined. Afterwards, the unique UIDs in the #particle data set of interests are collected (distinct_values). For all these micrographs, the distance between
the particles are calculated. However, only the minimum value is kept in the "distances" array, as this is most relevant for the analysis.

def calculate_distance(x1, y1, x2, y2):

  dx = x2 - x1
  dy = y2 - y1

  distance = (dy**2+dx**2)**0.5
  return distance

# Column to group on
group_column = 'location/micrograph_uid'

# Get distinct values in the group_column
distinct_values = J80[group_column].unique()

distances = []
for value in distinct_values:
  df1=J80.loc[J80['location/micrograph_uid'] == value]
  df2=J101.loc[J101['location/micrograph_uid'] == value]

  for i in range(len(df1)):
    sammlung = []
    for j in range(len(df2)):
      x1, y1 = df1.iloc[i]['x'], df1.iloc[i]['y']
      x2, y2 = df2.iloc[j]['x'], df2.iloc[j]['y']
      sammlung.append(calculate_distance(x1, y1, x2, y2))
    if len(sammlung) > 0:
      distances.append(np.min(sammlung))

# Create distance data frame
distance_df = pd.DataFrame({'distance': distances})

display(distance_df)

close_contacts = [x for x in distances if (x < 130) & (x > 70)]
no_contacts = [x for x in distances if (x >= 130) or (x <= 70)]

print(len(close_contacts))
print(len(no_contacts))
```

Supplementary Note 1: Python script for the calculation of the triangle-to-half-closed and triangle-to-wobbly-distances.

```
import pandas as pd
import numpy as np
import matplotlib.pyplot as plt

#Import the ITC data integrated with Nitpic
nitpic = pd.read_csv('240201_CA_MeCob_Data.nitpic', sep=None, engine="python")


# calculate the mean of the last data points as baseline
baseline = np.mean(nitpic["DH"][90:])
nitpic["base"]=nitpic["DH"]-baseline

#Calculate the cumulative heat of all injections
nitpic["cum"]=np.cumsum(nitpic["base"])

#Change the concentration values from mM to µM and correct for the active fraction determined by sedphat, use only first 91 data points
fit_data = pd.DataFrame(list(zip(nitpic["Xt"][0:90]*1000,nitpic["Mt"][0:90]*1000*0.921,nitpic["cum"][0:90])))

np.savetxt('CODHACS_MeCob.csv', fit_data, delimiter=' ')
```

Supplementary Note 2: Python script for the calculation of the cumulative heat for the CODH/ACS methyl cobinamide titration.

```
[task]
  task = fit
  data = equilibria

[mechanism]
  ACS + MC <===> MACS + C : K                    equil

[constants]
  K = 100??

[responses]
  MACS = -13??
[data]
offset = -1??
variable MC, ACS
plot titration
file ./Data/MeCob/CODHACS_MeCob.csv

[output]

directory ./Data/MeCob/1
[end]
```

Supplementary Note 3: Script used for analysis of the ITC data for CODH/ACS with methyl cobinamide by Dynafit4.

Supplementary Figures

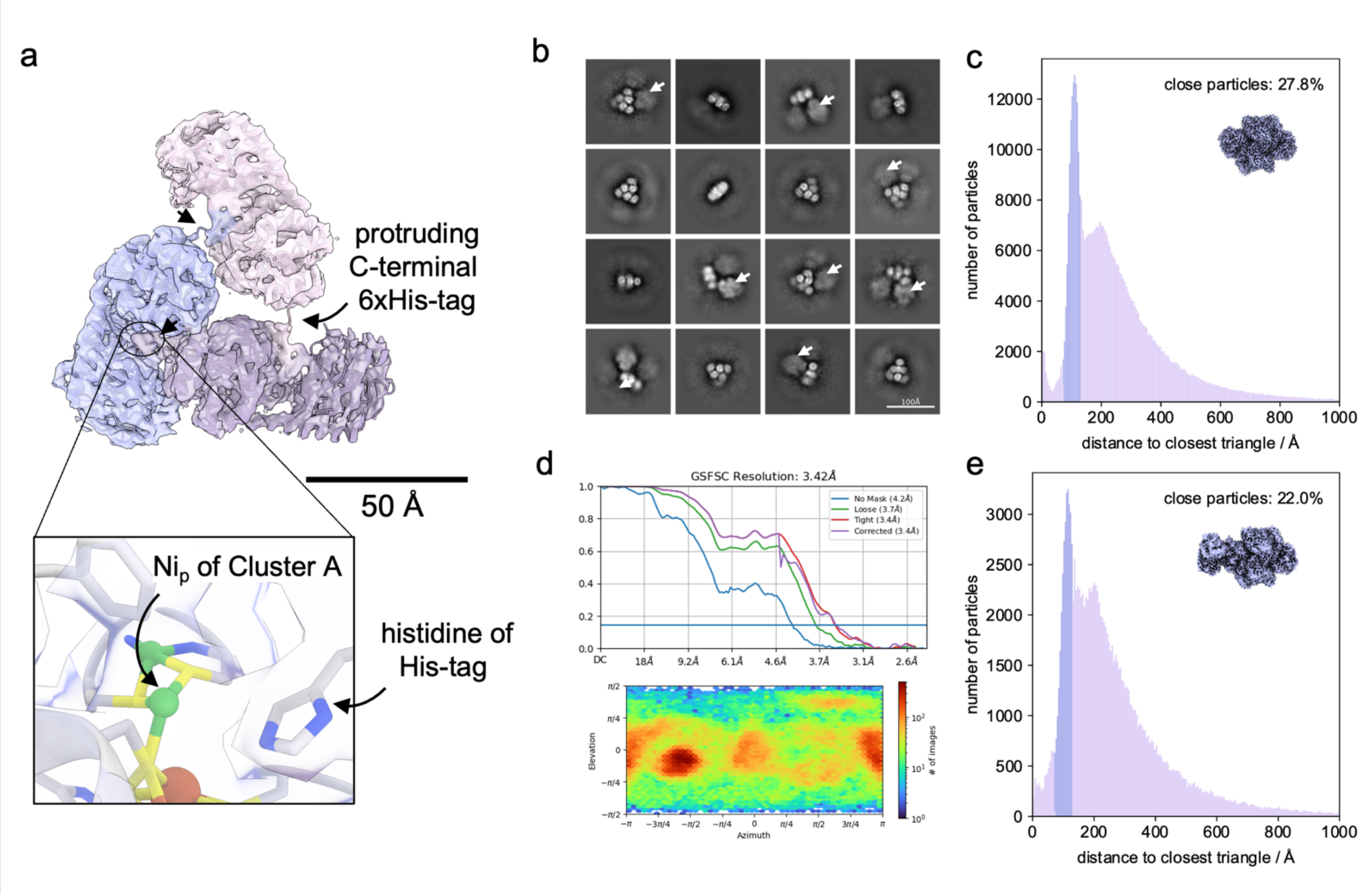

Supplementary Figure 1: The triangle species in the as-isolated data set. **a** Overview of the triangle species. The three copies of the ACS middle and N-terminal domain are colored pink, purple and lavender and shown in cartoon representation. The coulomb-potential map is shown as transparent surface around the atomic model and colored accordingly. The arrows mark the density for the C-terminal His-tag. The inset shows a close-up of cluster A with the His-tag of another ACS in its proximity. **b** representative 2D-class averages. The white arrows indicate the blurred density for the CODH/ACS core. **c** Histogram of the center-to-center-distance of the wobbly particles to the closest triangle particle. The contact distance (70 Å to 130 Å) is shaded in dark purple and the percentage of wobbly particles in that region in the plot. **d** Fourier-shell correlation and view distribution plot for the triangle species. **e** Histogram of the center-to-center-distance of the half-closed particles to the closest triangle particle (250 k particles after topaz picking, Extended Data Figure 1, calculated with the script shown in supplementary note 1). The contact distance (70 Å to 130 Å) is shaded in dark purple and the percentage of wobbly particles in that region in the plot.

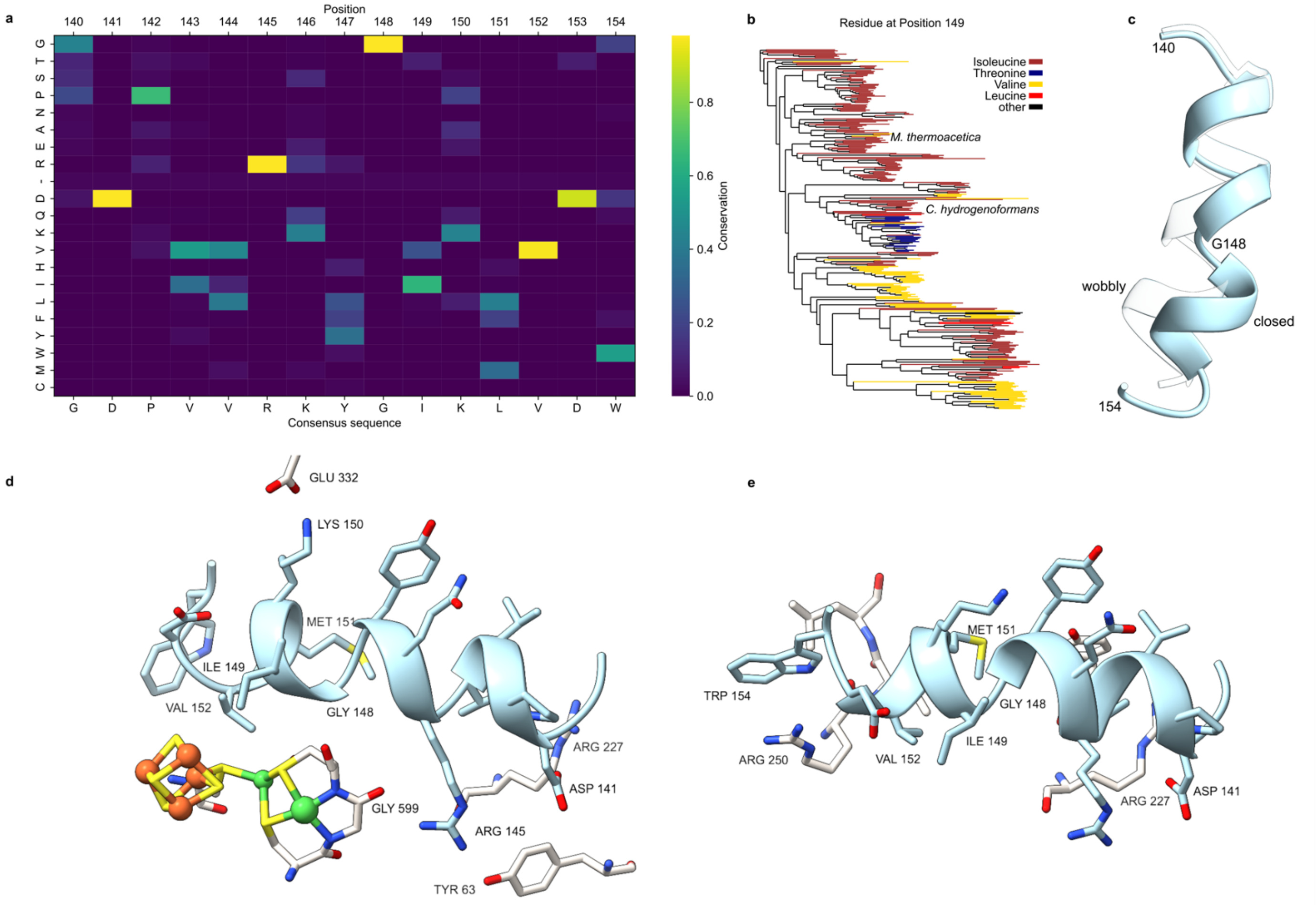

Supplementary Figure 2: Sequence and structure analysis of the gating helix. **a**, Sequence conservation of the gating helix in 334 ACS sequences. **b**, phylogenetic tree of those sequences (based on the Blosum62-based distance). The leaves are colored according to the residue found at position 149. **c**, comparison of the helix in the wobbly (transparent) and closed conformation (solid). Note the hinge introduced by the helix breaking G148. **d**, close-up of the gating helix (light blue) in the closed conformation in the reduced state. The color code follows Figure 1. Relevant residues are labeled. Major interaction are the salt-bridges between K150 and E332 and D141 and R227. Furthermore, there is a hydrogen-bond between R145 and G599 constituting the binding site of cluster A. **e**, close-up of the gating helix in the wobbly conformation in the reduced state. The only major non-hydrophobic interaction is here the π-π binding of R250 to W154.

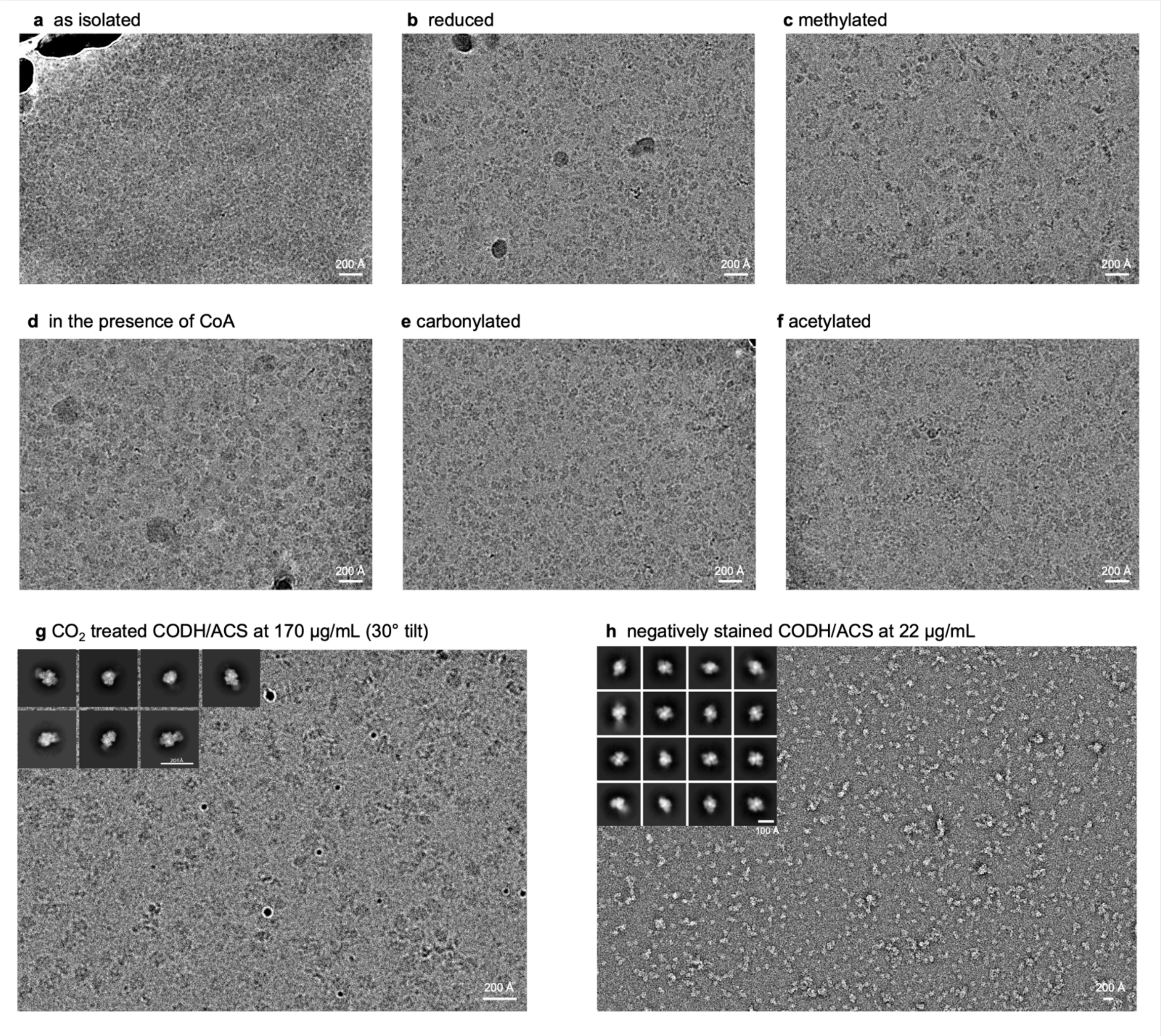

Supplementary Figure 3: Micrographs of CODH/ACS. Panels a to g show a typical micrograph of the data sets. All micrographs were recorded at a defocus of around -2 µm and low-pass filtered at 2Å. g, cryo-EM micrograph of CO_2 treated CODH/ACS at a lower particle density at 170 µg/mL (used for tilted data collection) . The inset shows obtained 2D-class averages showing wobbly, extended and half-closed species. fh typical micrograph of negatively stained CODH/ACS. The inset shows 2D class averages mainly showing the wobbly state. Details of the data sets can be found in Extended Data Table 3.

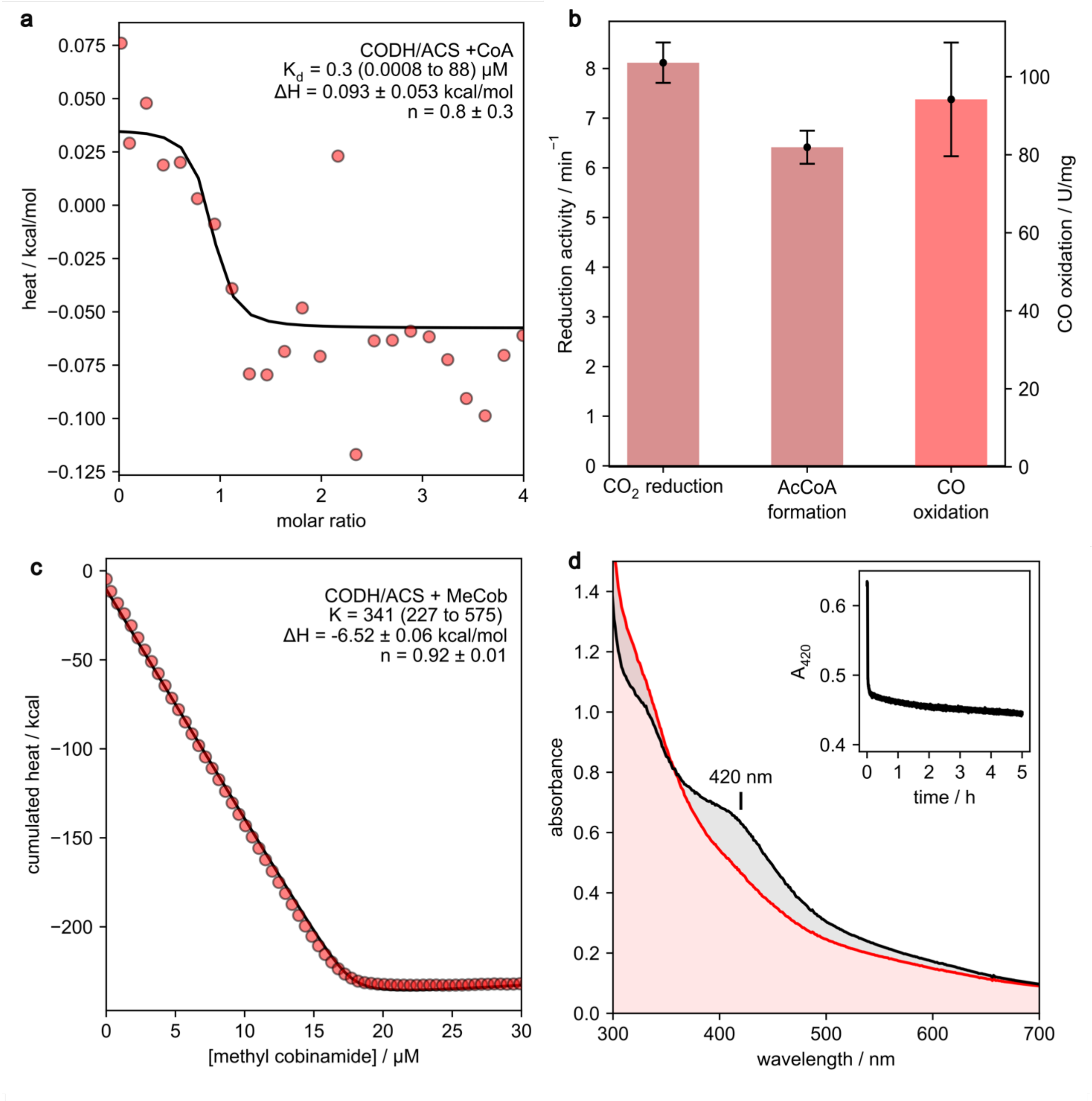

Supplementary Figure 4: Biochemical characterization of the CODH/ACS sample. ITC and reduction experiments were carried out at 25 °C in a 50 mM Mops pH 7.6, 150 mM NaCl buffer. For ITC experiments the buffer was further supplemented with 1 mM Ti(III)-EDTA. **a** Binding isotherm of CoA to CODH/ACS**.** 596 µM CoA were titrated to 20 µM CODH/ACS. The fit results are shown in the plot. **b** Enzymatic activity of the CODH/ACS batch used for cryo-EM experiments (average and standard deviation of 4 technical replicates (3 for CO_2 reduction). Activity measurements were performed at 50 °C in a 100 mM Mops pH 7.2 buffer supplemented with 3 mg/L carbonic anhydrase (CO_2 reduction and acetyl-CoA formation) or in a 50 mM Hepes pH 8, 2 mM DTT, 20 mM methyl viologen buffer saturated with CO (CO oxidation). **c** Binding isotherm of the methylation of CODH/ACS**.** 476 µM methyl cobinamide were titrated to 20 µM CODH/ACS. The fit results are shown in the plot. **d** UV-Vis spectrum of as isolated (black) CODH/ACS and after reduction with 1 mM Ti(III)-EDTA (red). The inset shows the time course of the absorbance at 420 nm.

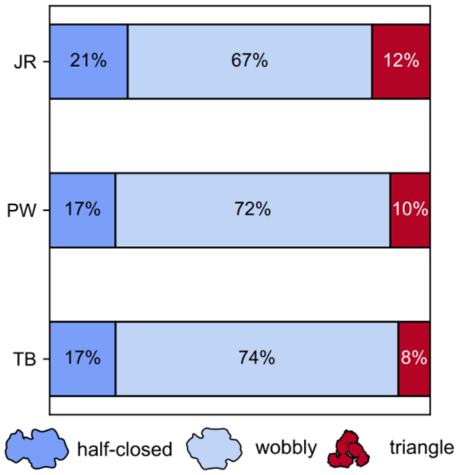

Supplementary Figure 5:Examiner bias analysis for the as isolated data set. The as isolated data set was examined independently by three different persons. All examiners followed the same initial strategy (Blob picking, template generation, template picking, 2D classification, 3D sorting). However, examiner JR used the Topaz neural-net picker for the triangle species. The standard deviations are 1.9 % (half-closed), 5.3 % (wobbly) and 3.5 % (triangle).

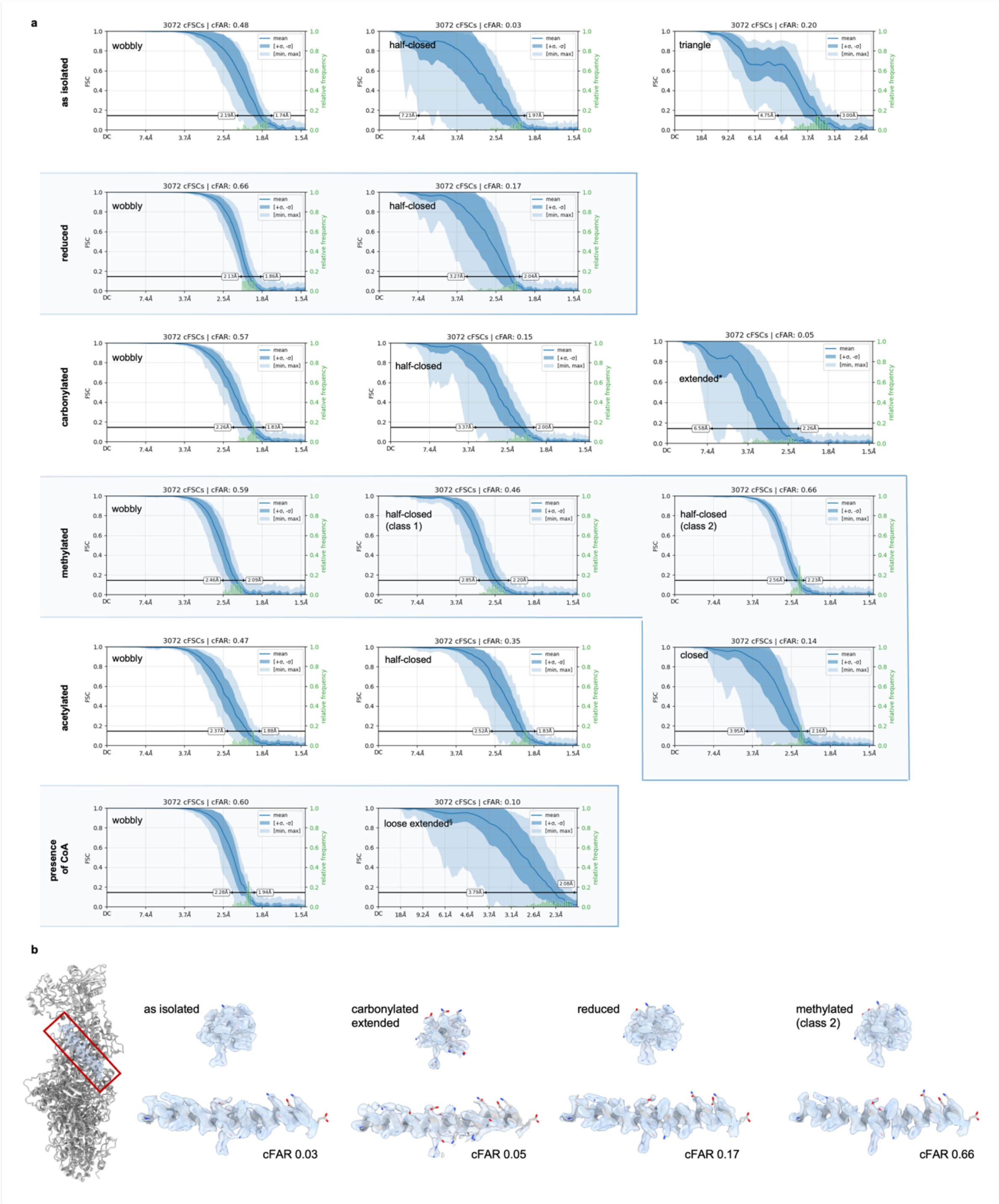

Supplementary Figure 6:Anisotropy analysis of all submitted maps. **a** 3DFSC plots were calculated with Cryosparc for 3072 viewing directions. For the extended state (*) and the loose extended state (§) we used the maps aligned on the CODH core for calculations. **b** visualization of anisotropy on the C-terminal helix of CODH. The positions of the helix in the half closed state is indicated on the left. The helix is shown from the top and side covering all viewing directions. B-factor sharpened maps are shown at 8 σ with different degrees of anisotropy with decreasing anisotropy from the left to the right: as isolated half closed state, carbonylated extended state, reduced half closed state and the half closed state (class 2) in the methylated sample.

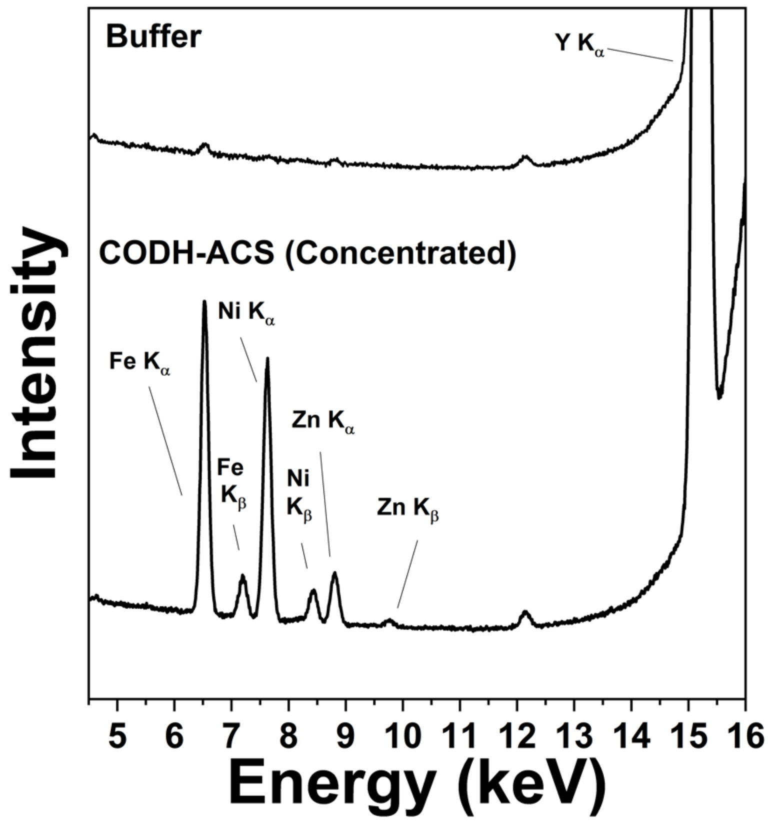

Supplementary Figure 7: Representative TXRF spectra of the CODH-ACS complex employed for structural studies for quantitative determination of the metal content. Metal content for the CODH-ACS complex is reported in Table S3. The top spectrum represents a spectrum of the buffer of the CODH-ACS complex, whereas the bottom spectrum represents the CODH-ACS complex (62.7 μM)

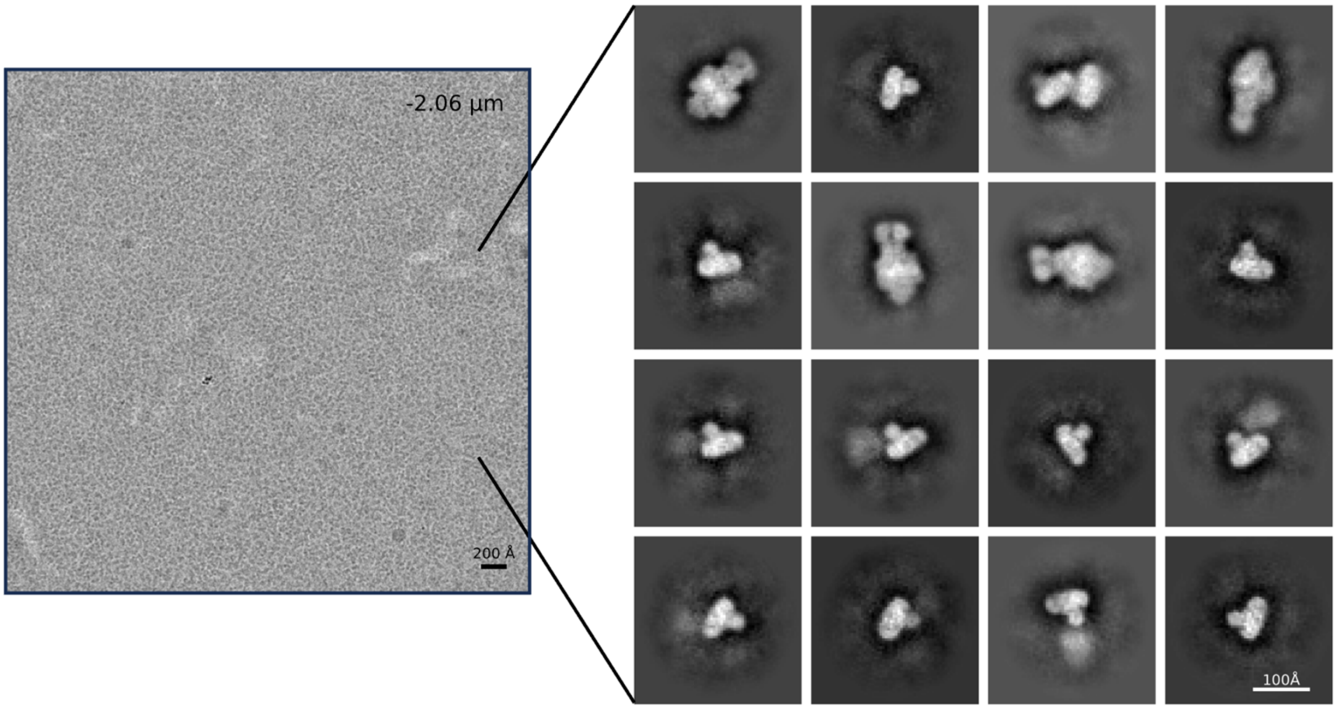

Supplementary Figure 8: Micrograph and 2D class averages for a data set of CODH/ACS (112 µg/mL) with methylated CoFeSP (135 µg/mL). Samples were diluted in 50 mM Mops pH 7.6, 150 mM NaCl, 1 mM Ti(III)-EDTA and incubated two hours before freezing in an anoxic atmosphere. Left, typical micrograph taken at a defocus of -2.06 µm and a dose of ~50 $e^{-}/Å^{2}$. Right, typical 2D class averages. While CODH/ACS classes of the half-closed state and CoFeSP alone is visible (e.g. lower two rows), no clear CODH/ACS:CoFeSP complex class was found. Details of the data sets can be found in Extended Data Table 3.

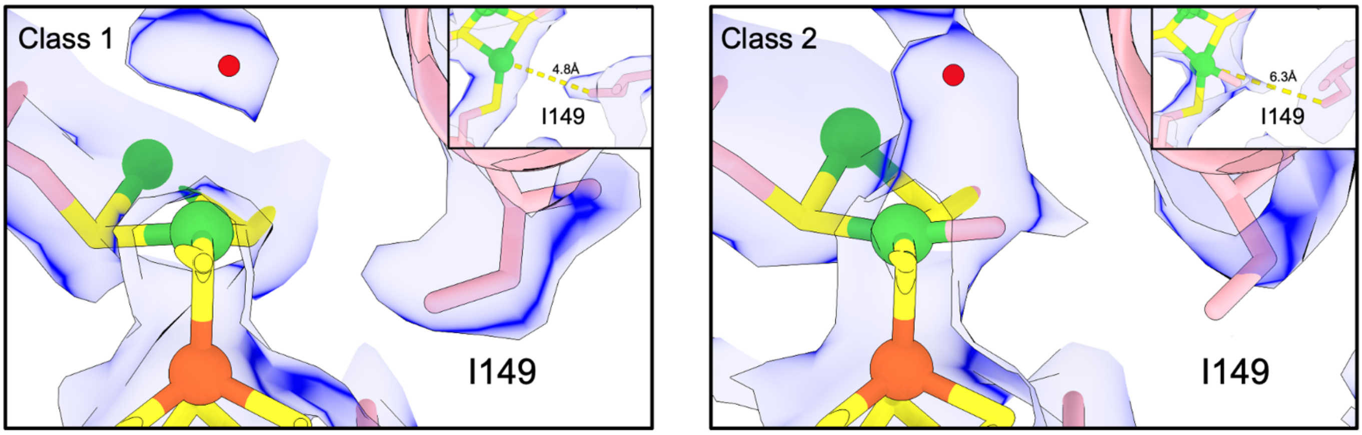

Supplementary Figure 9: Comparison of the two classes of the methylated half-closed state. The color code follows **Error! Reference source not found.**, the densities are shown at 5 σ. In class 1 no density for a methyl moiety in the axial position is visible, while in class 2 some density it is. This methyl-moiety might push I149 in the conformation seen in class 2.

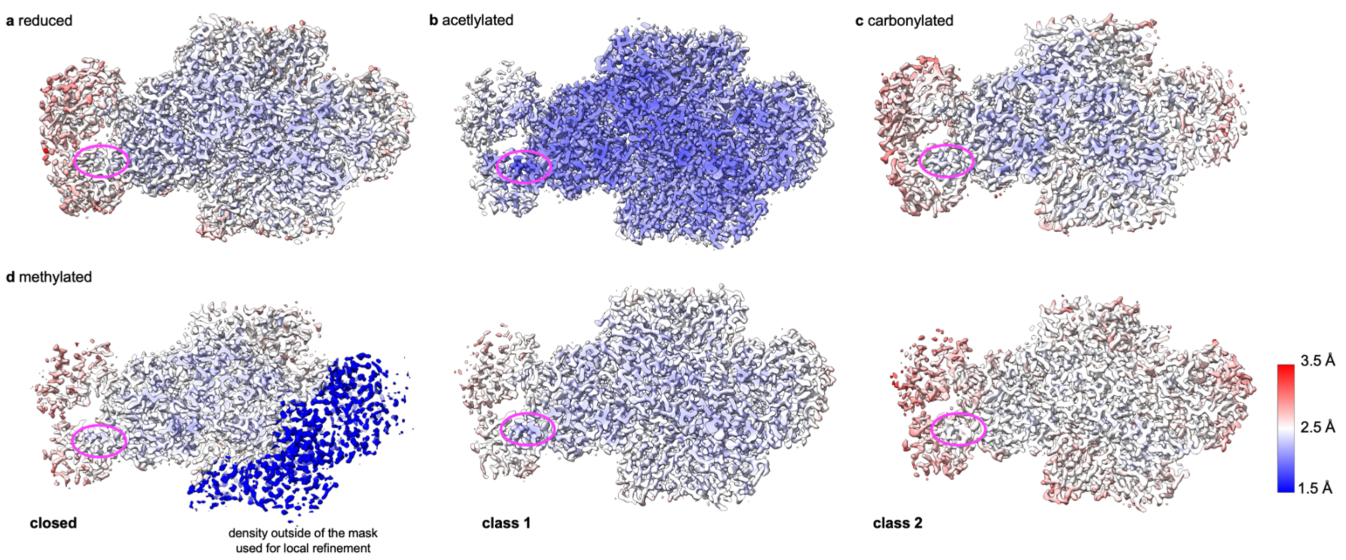

Supplementary Figure 10: Local resolution of the relevant half-closed and closed states. Maps are clipped at the plane containing cluster A. Cluster A's position is indicated by the pink ellipsoid. Maps are colored by their estimated local resolution (at FSC = 0.5) as indicated by the color bar. Panel a to d show the reduced, acetylated, carbonylated and methylated states, respectively.

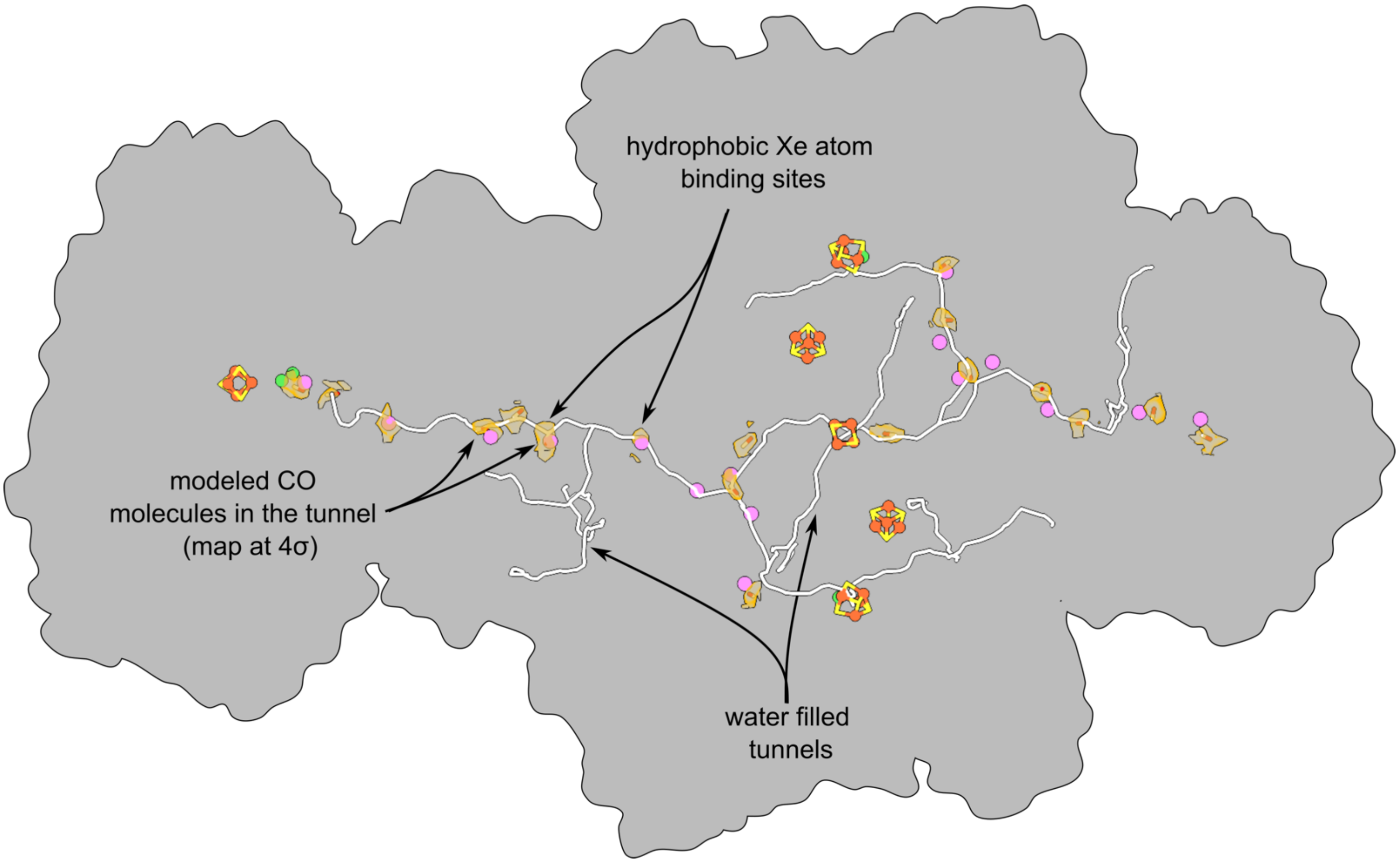

Supplementary Figure 11:Putative CO molecules in the tunnel in the half-closed state from the carbonylated data set. Densities in the hydrophobic tunnel system were modeled as CO (stick model, 17 CO molecules in the tunnel) The density map is shown at 4 σ 2 Å around the CO molecules. The Xenon binding sites (pink spheres) were taken from the structure of CODH/ACS of *M. thermoacetica* treated with Xenon (PDB ID 2Z8Y) The calculated tunnel system is shown as white line. Xenon atoms are shown as pink spheres, the coloring of CODH/ACS co-factors follows figure 2.

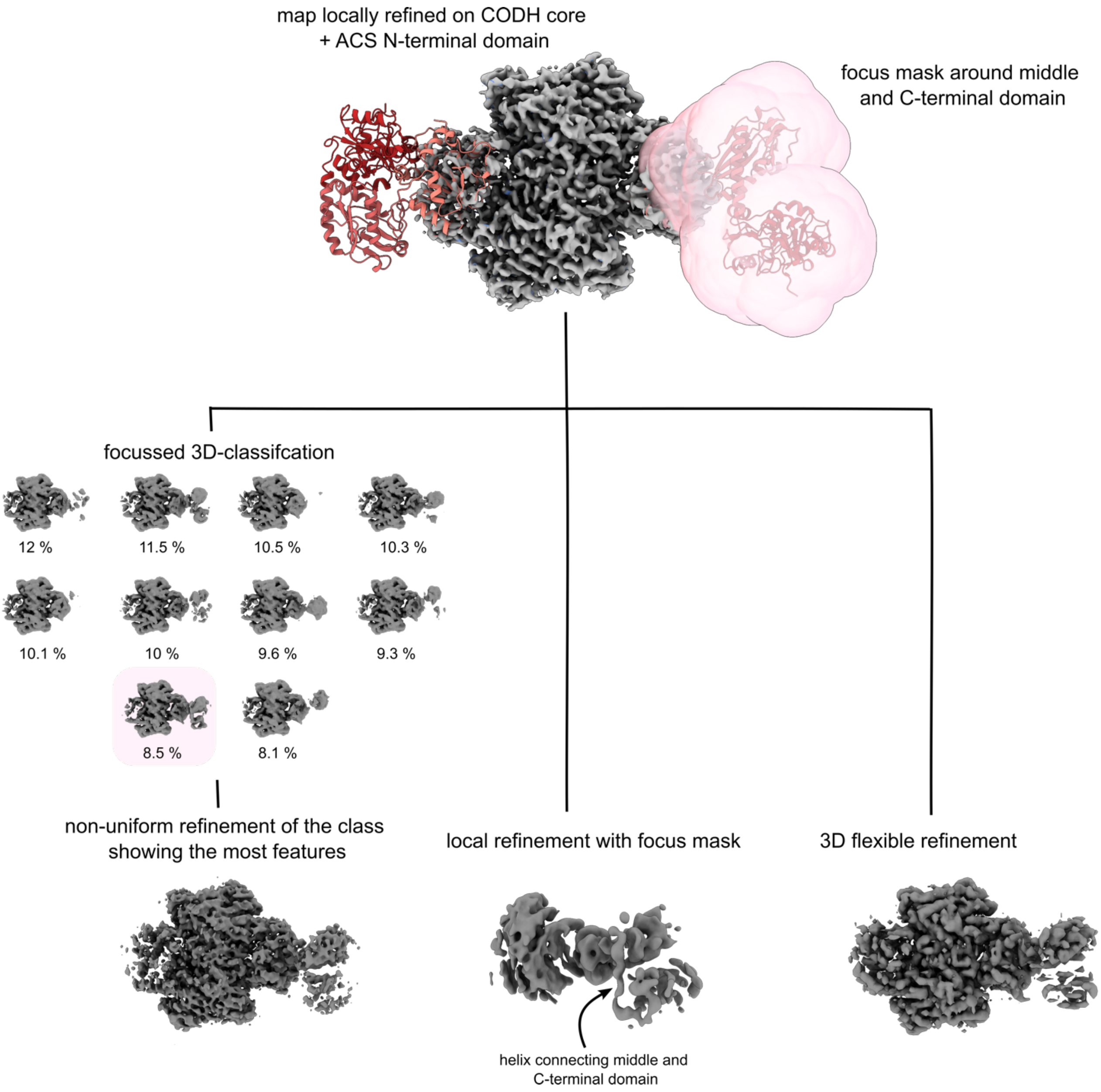

Supplementary Figure 12: Processing approaches to the flexibility of the extended species. Starting from the map locally refined on the CODH core (yielding the highest precision of particle alignment), we have chosen three processing strategies to tackle the problem of flexibility in the middle and C-terminal ACS domains. First, we generated a mask for the middle and C-terminal domain based on the AlphaFold3 model of ACS in complex with CoFeSP. With this map we performed either focussed 3D-classfication or a local refinement. None of the two approaches yielded high resolution maps of cluster A. 3D-flexible refinement gave a similar consensus map, which we have selected as our processing strategy as it reflects the conformational variability of ACS best.

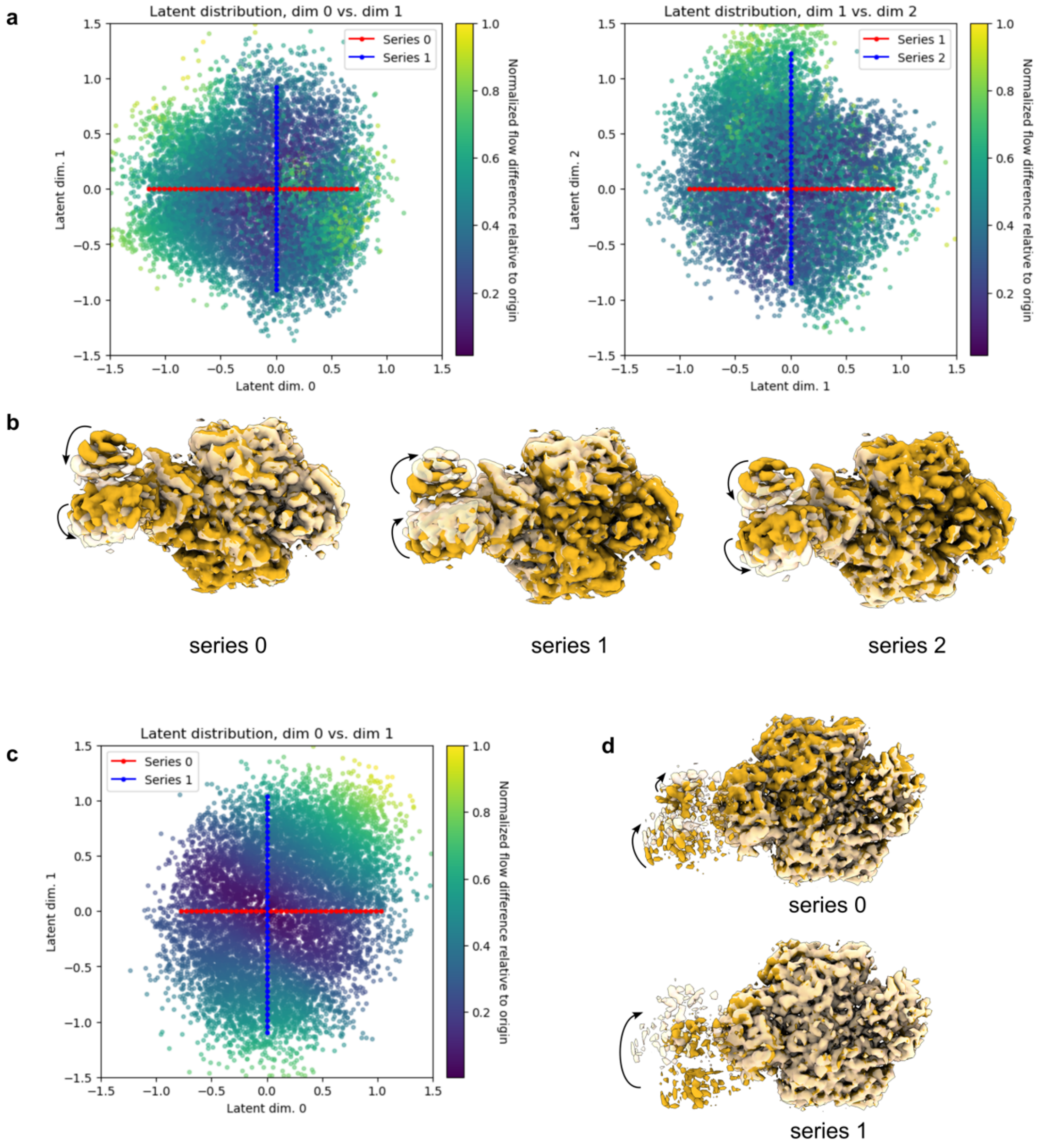

Supplementary Figure 13: Flexibility and calculated movements of the extended state in the carbonylated data set and the loose-extended state in the presence of CoA. **a**, Visualization of the three-dimensional latent conformational space of CODH/ACS in the extended state. Each point corresponds to a particle image and is colored according to its distance to the consensus volume. The red and blue line indicate slices through the latent space yielding the volume series shown in b. **b**, Volumes series from slices through the latent space for the extended state (4 σ). The solid map indicates the starting volume, and the transparent the end point. Movements are indicated by arrows. **c**, Visualization of the two-dimensional latent conformational space of CODH/ACS in the loose extended state. **d**, Volumes series from slices through the latent space for the loose extended state (5 σ). Depiction as in panel b.

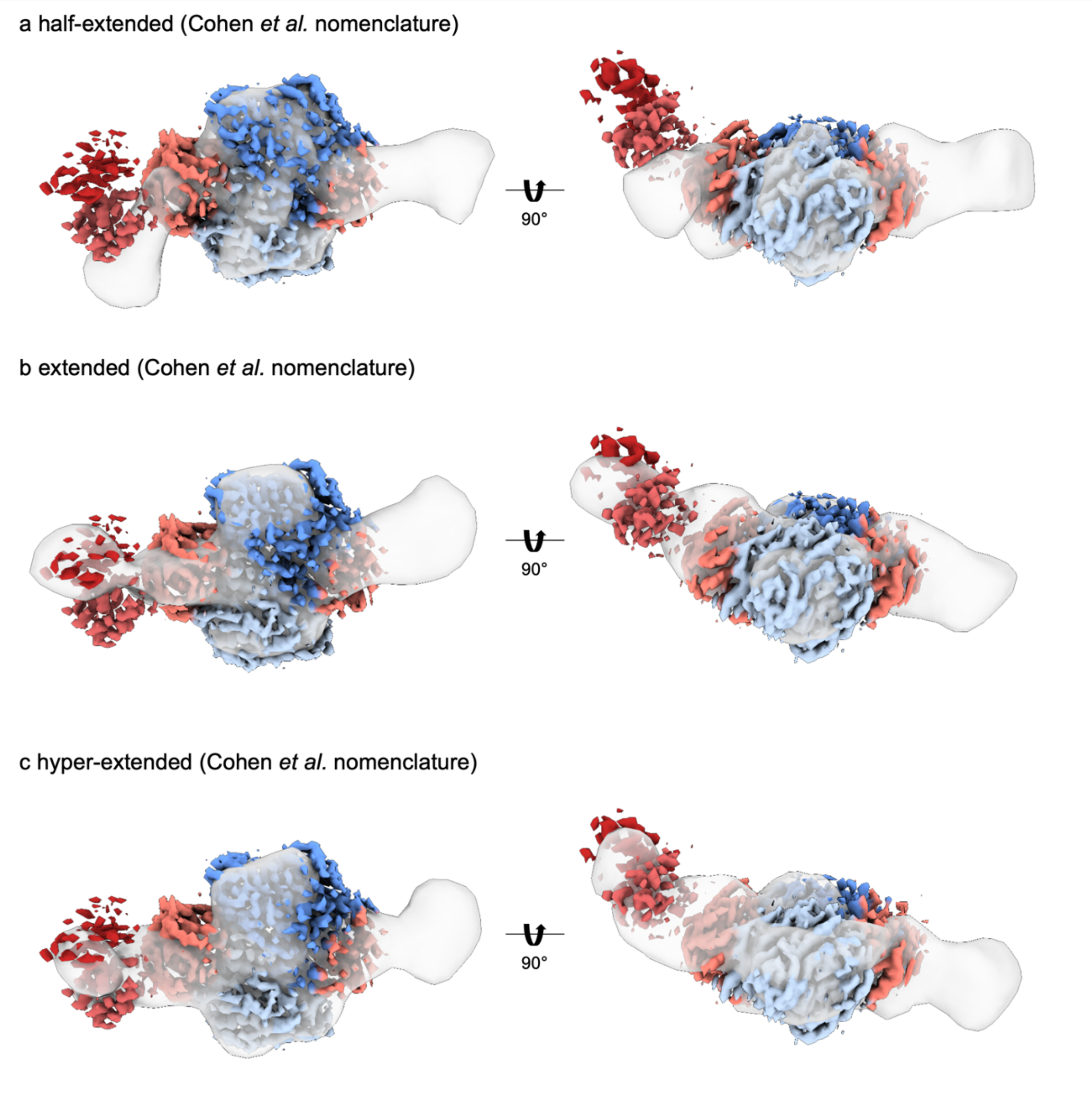

Supplementary Figure 14: Comparison of the extended state to those state found for *M. thermoacetica* CODH/ACS by negative-staining EM (Cohen *et al.*). **a** half-extended **b** extended and **c** hyper-extended state. The extended state that we found in the CO-data set is similar to the extended and hyper-extended maps. The color code follows Figure 2.

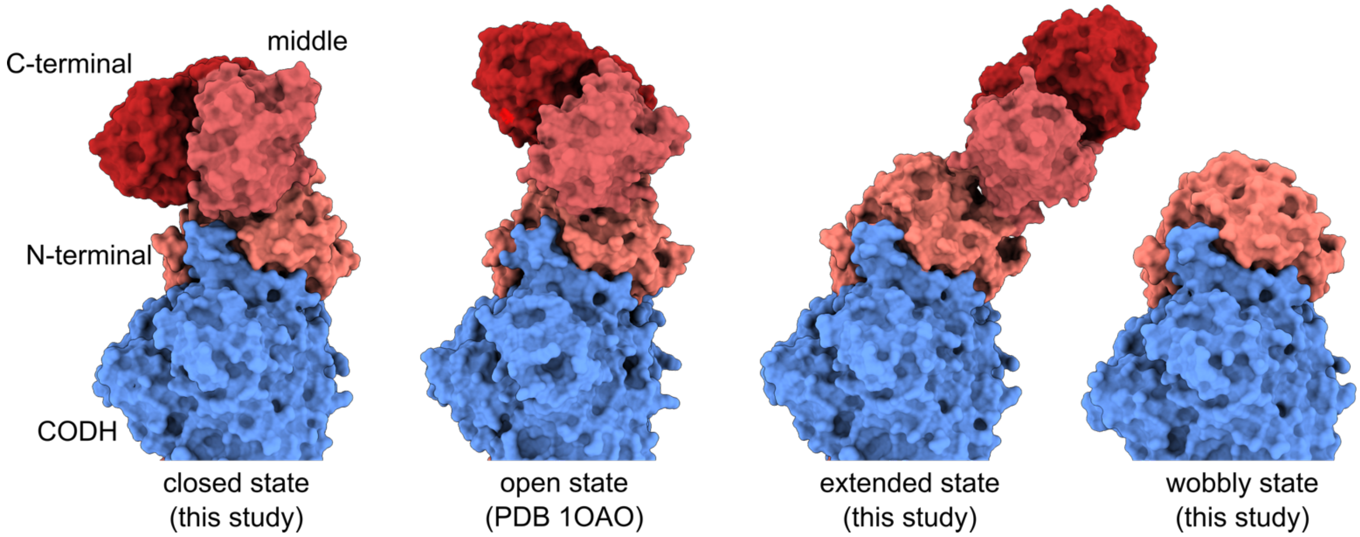

Supplementary Figure 15: Comparison of the states presented in this study to those identified before by x-ray crystallography. In the extended state the C-terminal domain is completely detached from the N-terminal domain. The open state (here shown for the M. thermoacetica CODH/ACS (PDB 1OAO) could be an intermediate state in the transition from the closed to the extended state. In the wobbly state the C-terminal and middle domain are not visible due to their flexibility. The color code follows Figure 2. One side of the CODH/ACS complex is shown in surface representation.

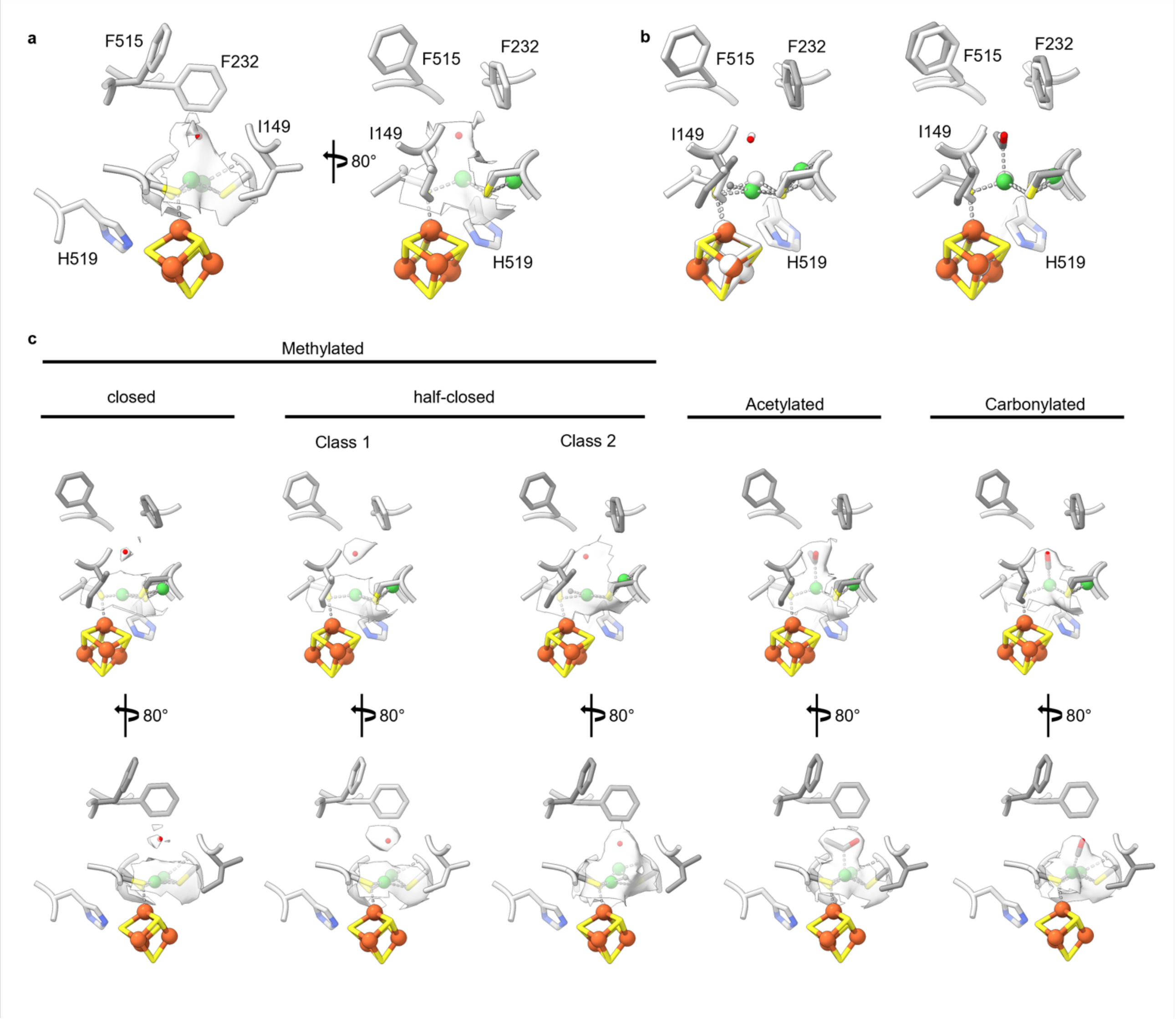

Supplementary Figure 16: Comparison of the environment of cluster A in the closed conformation. The color code follows Figure 1. Unsharpened maps are displayed at 6σ 3 A around Ni_p. a, Environment of the reduced state. b Overlay of the model of the reduced state (light grey) with two extrema. Left, methylated class 2. Note the movement of I149. Right, acetylated state. Note the slight movement of the phenylalanines. c, overview of the different states. The color code follows Figure 2.

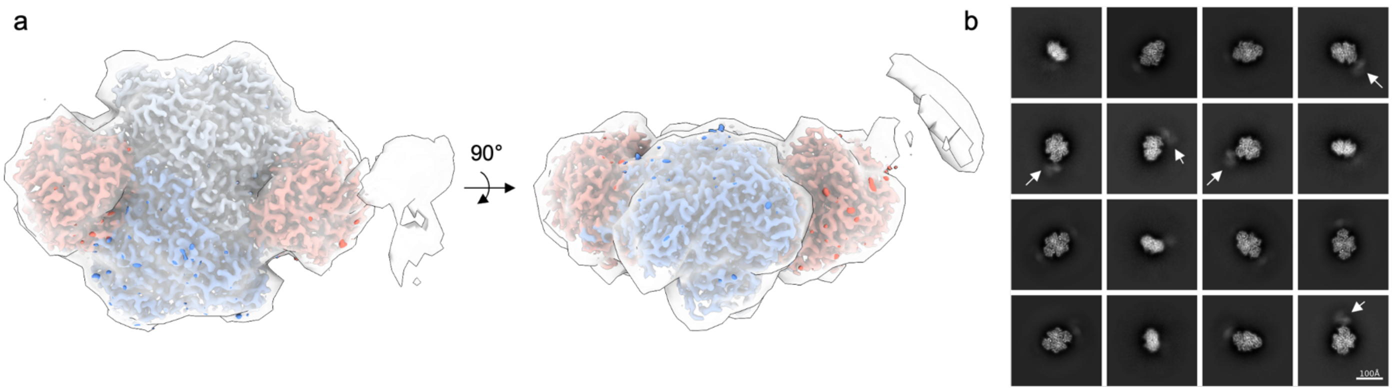

Supplementary Figure 17: Residual density above the N-terminal domain in the wobbly state from the acetylated data set. **a** Some density was only visible as shadows in 2D class averages. **b** Comparison of the high-resolution reconstruction (colored in the color code used in Figure 1, overlayed with the ab-initio reconstruction shown as grey transparent surface (limited to a resolution of 12 A) of a subset of wobbly particles showing a particularly strong density above the N-terminal domain. The densities are marked with white arrows.
